# Assessment of [^18^F]PI-2620 Tau-PET Quantification via Non-Invasive Automatized Image Derived Input Function

**DOI:** 10.1007/s00259-024-06741-7

**Published:** 2024-05-08

**Authors:** Maria Meindl, Artem Zatcepin, Johannes Gnörich, Maximilian Scheifele, Mirlind Zaganjori, Mattes Groß, Simon Lindner, Rebecca Schaefer, Marcel Simmet, Sebastian Roemer, Sabrina Katzdobler, Johannes Levin, Günter Höglinger, Ann-Cathrin Bischof, Henryk Barthel, Osama Sabri, Peter Bartenstein, Thomas Saller, Nicolai Franzmeier, Sibylle Ziegler, Matthias Brendel

**Affiliations:** 1 Department of Nuclear Medicine, LMU University Hospital, LMU Munich, Munich, Germany; 2 Department of Neurology, LMU University Hospital, LMU Munich, Munich, Germany; 3 German Center for Neurodegenerative Diseases (DZNE), Munich, Germany; 4 Munich Cluster for Systems Neurology (SyNergy), Munich, Germany; 5 Department of Neurology, Medizinische Hochschule Hannover, Hannover, Germany; 6 Department of Neurology, Technical University Munich, Munich, Germany; 7 Institute for Stroke and Dementia Research (ISD), Munich, Germany; 8 Department of Anesthesiology, LMU University Hospital, LMU Munich, Munich, Germany; 9 Department of Nuclear Medicine, University of Leipzig, Leipzig, Germany

**Keywords:** Alzheimer’s disease, Four-repeat tauopathies, Image derived input function, [^18^F]PI-2620 quantification, Tau

## Abstract

**Purpose:**

[^18^F]PI-2620 positron emission tomography (PET) detects misfolded tau in progressive supranuclear palsy (PSP) and Alzheimer’s disease (AD). We questioned the feasibility and value of absolute [^18^F]PI-2620 PET quantification for assessing tau by regional distribution volumes (V_T_). Here, arterial input functions (AIF) represent the gold standard, but cannot be applied in routine clinical practice, whereas image-derived input functions (IDIF) represent a non-invasive alternative. We aimed to validate IDIF against AIF and we evaluated the potential to discriminate patients with PSP and AD from healthy controls by non-invasive quantification of [^18^F] PET.

**Methods:**

In the first part of the study, we validated AIF derived from radial artery whole blood against IDIF by investigating 20 subjects (ten controls and ten patients). IDIF were generated by manual extraction of the carotid artery using the average and the five highest (max5) voxel intensity values and by automated extraction of the carotid artery using the average and the maximum voxel intensity value. In the second part of the study, IDIF quantification using the IDIF with the closest match to the AIF was transferred to group comparison of a large independent cohort of 40 subjects (15 healthy controls, 15 PSP patients and 10 AD patients). We compared V_T_ and V_T_ ratios, both calculated by Logan plots, with distribution volume (DV) ratios using simplified reference tissue modelling and standardized uptake value (SUV) ratios.

**Results:**

AIF and IDIF showed highly correlated input curves for all applied IDIF extraction methods (0.78 < r < 0.83, all *p* < 0.0001; area under the curves (AUC): 0.73 < r ≤ 0.82, all *p* ≤ 0.0003). Regarding the V_T_ values, correlations were mainly found between those generated by the AIF and by the IDIF methods using the maximum voxel intensity values. Lowest relative differences (RD) were observed by applying the manual method using the five highest voxel intensity values (max5) (AIF vs. IDIF manual, avg: RD = -82%; AIF vs. IDIF automated, avg: RD = -86%; AIF vs. IDIF manual, max5: RD = -6%; AIF vs. IDIF automated, max: RD = -26%). Regional V_T_ values revealed considerable variance at group level, which was strongly reduced upon scaling by the inferior cerebellum. The resulting V_T_ ratio values were adequate to detect group differences between patients with PSP or AD and healthy controls (HC) (PSP target region (globus pallidus): HC vs. PSP vs. AD: 1.18 vs. 1.32 vs. 1.16; AD target region (Braak region I): HC vs. PSP vs. AD: 1.00 vs. 1.00 vs. 1.22). V_T_ ratios and DV ratios outperformed SUV ratios and V_T_ in detecting differences between PSP and healthy controls, whereas all quantification approaches performed similarly in comparing AD and healthy controls.

**Conclusion:**

Blood-free IDIF is a promising approach for quantification of [^18^F]PI-2620 PET, serving as correlating surrogate for invasive continuous arterial blood sampling. Regional [^18^F]PI-2620 V_T_ show large variance, in contrast to regional [^18^F]PI-2620 V_T_ ratios scaled with the inferior cerebellum, which are appropriate for discriminating PSP, AD and healthy controls. DV ratios obtained by simplified reference tissue modeling are similarly suitable for this purpose.

**Supplementary Information:**

The online version contains supplementary material available at 10.1007/s00259-024-06741-7.

## Introduction

The tracer [^18^F]PI-2620 shows high potential for positron emission tomography (PET) imaging of Alzheimer’s disease (AD) [1], the 4R-tauopathies progressive supranuclear palsy (PSP) [2], and corticobasal syndrome (CBS) [3]. It is investigationally used to support the diagnosis of AD and PSP at tertiary centers [1]. Pathological aggregation of hyperphosphorylated microtubule-associated tau is characterized by equal amounts of 3- and 4-repeat isoforms in neurons of patients with AD and by predominant 4-repeat isoforms in neurons and glial cells of patients with PSP [4]. In this regard, differences in tau isoforms have an impact on [^18^F]PI-2620 quantification [5], potentially due to distinct binding capacities [6].

Hitherto, [^18^F]PI-2620 quantification was predominantly performed using the cerebellar grey matter as a reference tissue for kinetic modeling [3, 4] or standardized uptake value (SUV) ratios of late static frames [7]. However, absolute quantification of tracer binding by estimating regional distribution volumes (V_T_) could be important, since topological heterogeneity of tau aggregation can also affect reference regions like the inferior cerebellum [8]. Absolute PET quantification requires an accurate knowledge of [^18^F]PI-2620 concentration in arterial blood as a function of time. Arterial blood derived input functions (AIF) reflect the gold-standard using continuous sampling of blood from the radial artery. However, this is an uncomfortable invasive procedure that involves personnel effort and burden to the patients. A non-invasive alternative is the image derived input function (IDIF), which is directly obtained from the PET images.

In this work, we present methods for generating completely blood-free [^18^F]PI-2620 IDIF by manual and automated extraction of the carotid artery, which were validated against AIF. In addition, we investigated whether patients with AD and PSP can be distinguished from healthy controls using regional V_T_ and V_T_ ratios calculated with the obtained input functions, compared to reference tissue modeling and late-phase ratios.

## Materials and methods

### Study design

#### Part I of the study

**Fig. 1:**
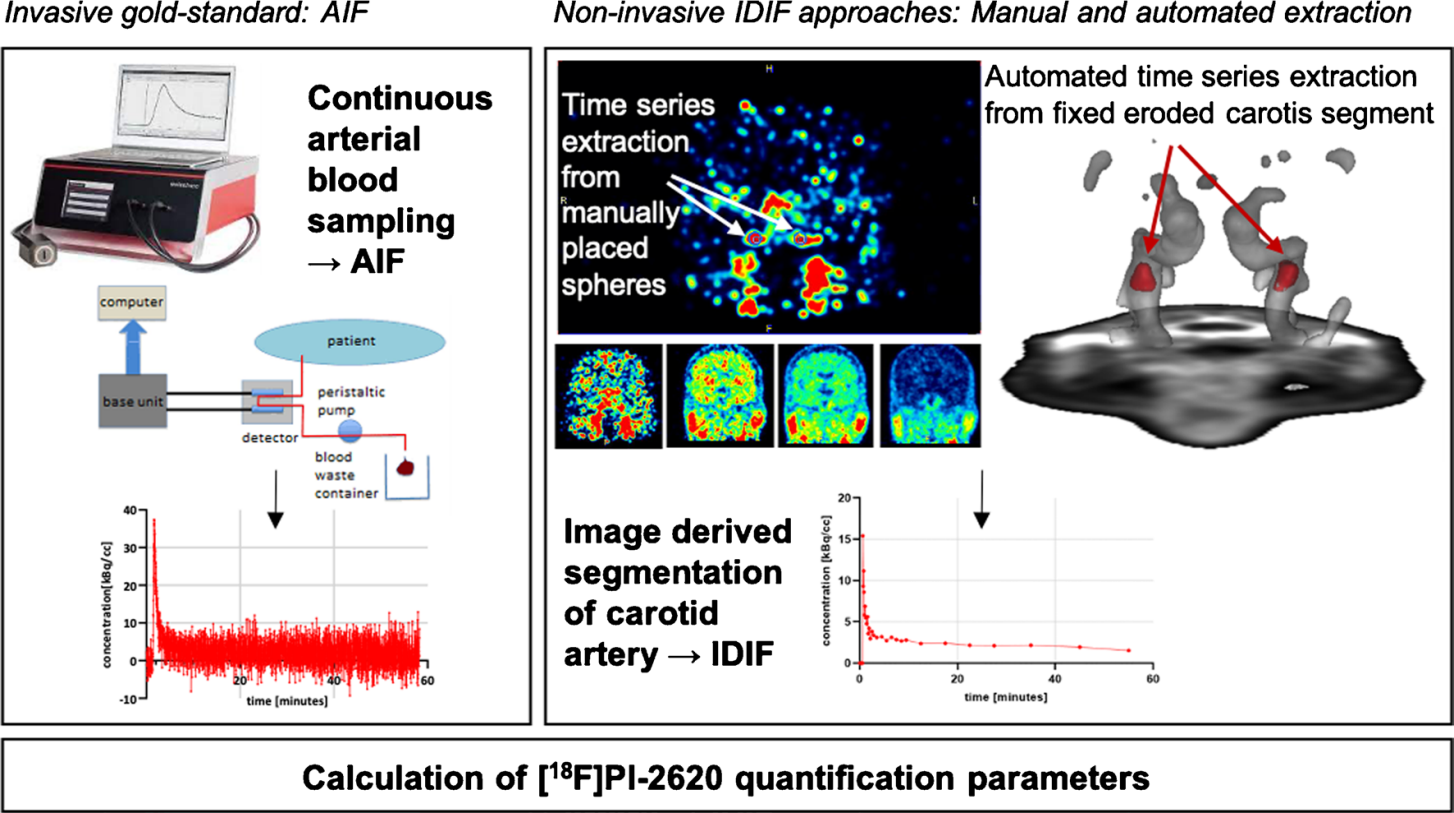
Obtaining the input functions by continuous sampling of whole blood from the radial artery (left) and by manual (middle) and automated (right) extraction of the PET signal from the carotid artery. Calculation and comparison of [^18^F]PI-2620 quantification parameters such as V_T_, V_T_ ratio, DV ratio and SUV ratio values using AIF and IDIF

In the first part of the study, we performed arterial blood sampling in 20 subjects and validated the resulting AIF against four different IDIF generated by manual and automated extraction of the PET signal from the carotid artery (Fig. 1). As a result, the IDIF with the closest match to the AIF was determined.

#### Part II of the study

In the second part of the study, we performed IDIF quantification in 40 subjects using the IDIF protocol which showed the highest correlation with AIF. The subjects belonged to three different groups, namely healthy controls, PSP patients and AD patients. The obtained quantification parameters were used to evaluate whether they were suitable to differentiate between the groups.

### Subjects

#### Part I of the study

Arterial blood sampling was performed as part of an ongoing study protocol in patients with PSP and healthy controls (EudraCT-Nr.: 2021–000201-24, ethics committee of the LMU Munich: approval ID 21–0170) and in an observational study in multiple diseases (DRKS00016920, ethics committee of the LMU Munich: approval IDs 17–569 and 19–022). Per 12/2023 eight healthy controls, ten patients with PSP and two disease controls (one patient with Parkinson disease (PD) and one patient with frontotemporal dementia (FTD)) were included (Table 1).

**Table 1:** Subject characteristics

	Healthy controls	PSP patients	Disease controls
Part I of the study			
Total number	8	10	2
Subgroups	NA	PSP-RS (n = 7), PSP-non-RS (n = 3)	PD (n = 1), FTD (n = 1)
Age, mean	70 years	71 years	66 years
Sex	female (n = 4), male (n = 4)	female (n = 5), male (n = 5)	male (n = 2)
Disease duration, mean	NA	39 months	NA
Part II of the study			
Total number	15	15	10
Subgroups	NA	PSP-RS, (n = 12), PSP-non-RS (n = 3)	AD-DEM (n = 8), AD-MCI (n = 2)
Age, mean	69 years	69 years	67 years
Sex	male (n = 8), female (n = 7)	male (n = 7), female (n = 8)	male (n = 2), female (n = 8)
Disease duration, mean	NA	54 months	32 months

#### Part II of the study

In the second part of the study, healthy controls, patients with PSP, and patients with AD were randomly selected from the ongoing observational study. 15 healthy controls were included together with 15 patients with probable or possible PSP according to current diagnostic criteria [9] and 10 patients with biologically defined typical AD (A + T + N +) [10] (Table 1). The ATN criteria (concerning the pathological processes ß **a**myloid deposition (A), pathologic **t**au (T) and **n**eurodegeneration (N)) were defined on PET images in a clinical routine setting via visual inspection of late phase images (90–110 min post-injection). Amyloid rating was additionally supported by semi-quantitative analysis using HERMES Gold software (Hermes Medical Solutions AB, Stockholm, Sweden), but we assured that no borderline cases were included. Thus, all A + cases were positive based on a visual amyloid PET read (tracer: [^18^F]Flutemetamol (FMM) or [^18^F]Florbetaben (FBB), median administered activity: 182 ± 11 MBq, same PET scanner as used for tau-PET). Visual rating of tau-positivity was performed with adaption of the FDA approach for [^18^F]Flortaucipir to [^18^F]PI-2620. To this end, [^18^F]PI-2620 images (30–60 min) [1] were scaled by the cerebellum and visually inspected by trained readers. The N status was examined based on the early phase of amyloid [11–14] using Minoshima projections as commonly used for [^18^F]FDG PET. Furthermore, all images were in parallel inspected via the brain tool of HERMES Brass software (Hermes Medical Solutions AB, Stockholm, Sweden). Z-score deviation of more than two in AD-typical regions and an AD-like pattern were applied as semiquantitative visually guided criteria.

### PET imaging

[^18^F]PI-2620 was synthesized as previously described [4]. The administered activity ranged between 156 and 223 MBq (median administered activity: 189 MBq), applied as a slow (10 s) intravenous bolus injection.

PET imaging was performed in a full dynamic setting (scan duration: 0–60 min post-injection) using a Siemens Biograph True point 64 PET/CT (Siemens, Erlangen, Germany) or a Siemens mCT (Siemens, Erlangen, Germany). The dynamic brain PET data were acquired in list-mode over 60 minutes and reconstructed into 35 time frames (12 × 5 s, 6 × 10 s, 3 × 20 s, 7 × 60 s, 4 × 300 s and 3 × 600 s) using a 336 × 336 × 109 matrix (voxel size: 1.02 × 1.02 × 2.03 mm^3^) and the built-in 3-dimensional ordered subset expectation maximization (OSEM) algorithm with 4 iterations, 21 subsets and a 5 mm full-width-at-half-maximum Gaussian filter on the Siemens Biograph and with 5 iterations, 24 subsets and a 5 mm full-width-at-half-maximum Gaussian filter on the Siemens mCT. A CT served for attenuation correction (tube voltage: 120 kV, tube current: 33 mA, pitch: 1.5, rotation time: 0.5 s). As scatter correction, single scatter simulation was used.

### Input function

#### Part I of the study

##### Arterial input function

AIF were obtained by continuous sampling of whole blood from the radial artery using the Swisstrace Blood Sampling System (Swisstrace, Menzingen, Switzerland) (Fig. 1). The blood flow was controlled by a peristaltic pump (0–5 min post-injection: 300 ml/min, 6–20 min post-injection: 150 ml/min, 21–60 min post-injection: 20 ml/min). The measured activity concentration was decay corrected. The cross-calibration of the external detector of the blood sampling system, the dose calibrator and the PET scanner was routinely checked.

##### Image derived input function

IDIF were generated by manual and automated extraction of the PET signal from the carotid artery over the 60-minute dynamic PET scan. There was an initial quality control for all PET images, also with regard to motion. PET images which showed too much motion (> 10 mm) were excluded [15]. This was the case for two subjects. The included PET images showed an average movement in x, y and z direction of 0.24 mm, 0.35 mm, 1.61 mm.

For manual extraction, the blood activity concentration in the bilateral carotid artery was detected in early frames of the dynamic PET images (usually frame 1 to 7), and spheres with a diameter of 5.0 mm were placed as volumes of interest (VOI) in the pars cervicalis of the internal carotid artery prior to entering the pars petrosal using PMOD version 4.2 (PMOD Technologies, Zürich, Switzerland) (Fig. 1). The activity concentration over time was calculated with the average and the five highest (max5) voxel intensity values (similar approach see [16]) of the VOI.

For automated extraction of carotid artery SUV time series, dynamic PET images were first motion corrected using the implemented motion correction tool of PMOD (i.e. rigid alignment of subsequent frames) and averaged. The resulting mean PET image was then warped to Montreal Neurology Institute (MNI) space via the 30–60 minutes summation image, using a custom in-house [^18^F]PI-2620 MNI template obtained by the PNEURO pipeline [17], via a high dimensional non-linear warping algorithm implemented in the Advanced Normalization Tools Software (ANTs) package.

Independent component analysis (ICA) with a pre-defined 10 component solution was applied to the native space dynamic PET image to parcellate the image into variance components that represent maps of temporally correlated voxels. The underlying rationale is that voxels belonging to the carotid artery should show a highly temporally correlated SUV signal across the dynamic scan, which should be identifiable using ICA. The resulting component maps were warped to MNI space using the ANTs-derived high-dimensional warping parameters and matched against a custom in-house carotid artery template in MNI space using spatial correlation to extract a subject-specific carotid component. The subject-specific carotid component in the MNI space was then automatically masked using a binary image that restricts the carotid artery to a segment in the upper part of the pars cervicalis, in line with the manual approach described above. Lastly, the masked subject-specific carotid image was warped back to native space using the ANTs derived warping parameters with nearest-neighbour interpolation to maintain a binary image. This image was further eroded using FSL to eliminate voxels close to the vessel walls, which may confound the carotid signal. The eroded binary carotid image was then applied to the native space dynamic PET image to extract the activity-time series (average and maximum value) across the 60 minutes scanning duration within the segment that corresponds to the manually selected volume (Fig. 1).

To compare the input functions, the activity concentrations obtained from continuous blood sampling were averaged over intervals corresponding to the frame durations of the PET images. Furthermore, the delay between the arrival of radioactivity in the radial artery and the carotid artery was considered by matching the IDIF peak to the peak of the AIF.

#### Part II of the study

##### Image derived input function

IDIF were generated by the manual method with the five highest voxel intensity values (max5).

### Quantification Parameters

#### Part I of the study

Volume of distribution (V_T_) images were calculated with the AIF and IDIF using Logan plots [18], which assume that the data become linear after an equilibration time t*. t* was fitted based on the maximum error criterion, which indicates the maximum relative error between the linear regression and the Logan-transformed measurements in the segment starting from t*. The maximum error was set to 10%. The percent masked pixels were set to 0%. The Putamen, which was defined by manual placement of a VOI (sphere with a diameter of 10 mm), served as tissue region.

All images were transformed to MNI space using the established [^18^F]PI-2620 PET template [7]. Automatized brain normalization settings in PMOD included nonlinear warping, 8 mm input smoothing, equal modality, 16 iterations, frequency cutoff 3, regularization 1.0, and no thresholding. Using the mean voxel value of a VOI placed in the inferior cerebellum as the scaling factor, V_T_ ratio images were calculated.

Average V_T_ and V_T_ ratio values were obtained in 9 PSP target regions, predefined by the atlas of basal ganglia [19], the Brainnetome atlas [20], and the Hammers atlas [21], based on earlier autopsy data [22]: globus pallidus (internus and externus), putamen, subthalamic nucleus, substantia nigra, dorsal midbrain, dentate nucleus, dorsolateral prefrontal cortex (DPFC), and medial prefrontal cortex (MPFC).

#### Part II of the study

In addition to V_T_ images, distribution volume (DV) and SUV images were calculated. For computing DV images, simplified reference tissue modeling (SRTM2) was performed as implemented in the freely available QModeling toolbox (for detailed methods see [23]). Using the mean voxel value of a VOI placed in the inferior cerebellum as the scaling factor, V_T_ ratio, DV ratio and SUV ratio images were calculated. All images were transformed to MNI space.

Average V_T_, V_T_ ratio, DV ratio and SUV ratio values were obtained in the 9 PSP target regions. In addition, average V_T_, V_T_ ratio, DV ratio and SUV ratio values were obtained in Braak regions [24]. The regional V_T_, V_T_ ratio, DV ratio and SUV ratio values were additionally transformed into z-score values by subtracting the mean of the healthy controls from each value and then dividing by the standard deviation of the healthy controls (z-score = (value – mean) / standard deviation).

### Statistics

GraphPad Prism version 9.1.2 (226) (GraphPad Software, San Diego, United States) was used for statistical testing. P values less than 0.05 were considered significant (* *p* < 0.05, ** *p* < 0.01, *** *p* < 0.001, **** *p* < 0.0001). Before all t-tests, normality tests (D’Agostino & Pearson test, Anderson–Darling test, Shapiro–Wilk test, Kolmogorov–Smirnov test) were performed using QQ plots. The radioactivity concentrations and the area under the curves (AUC) of the AIF and the IDIF were compared using a paired two-tailed t-test, Pearson correlation coefficients r, coefficients of determination r^2^ and a repeated measures ANOVA. Coefficients of variation (CoV) were estimated for regional V_T_ and V_T_ ratio values displayed as mean ± standard deviation and compared by a paired two-tailed t-test. Correlations between regional V_T_ values calculated with the AIF and the IDIF were examined using Pearson correlation coefficient r. Regional and overall V_T_ and V_T_ ratio values of patients with PSP and healthy or disease controls were compared by using an unpaired two-tailed t-test. Comparisons between V_T_, V_T_ ratio, SUV ratio and DV ratio values were done using one way ANOVA, Pearson correlation coefficients r and Cohen’s D.

## Results

### Head-to-head Comparison of AIF and IDIF

**Fig. 2:**
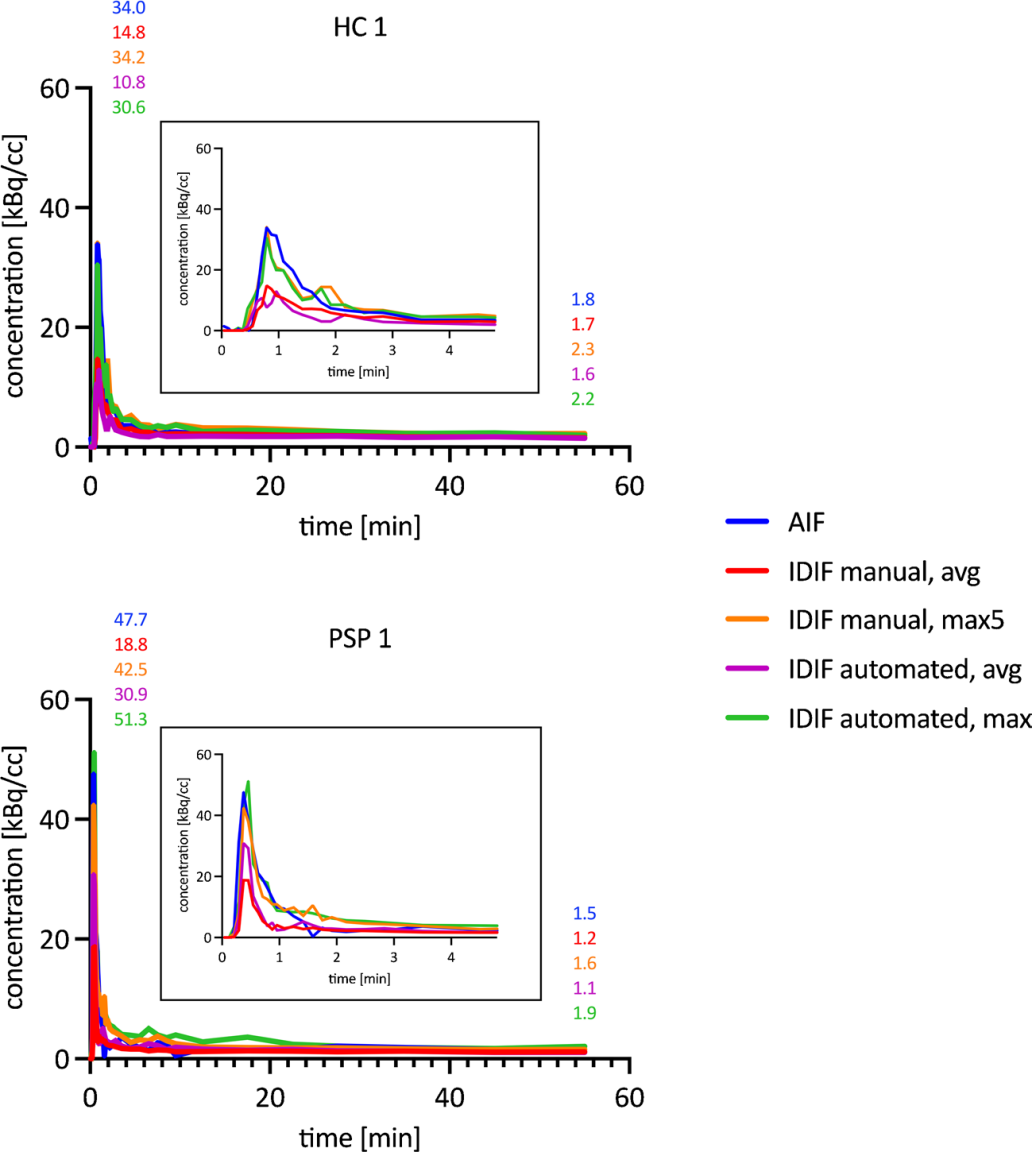
Comparison of AIF and IDIF generated with the manual and automated methods on one of the healthy controls and one of the PSP patients. The input functions of the other subjects are presented in Supplemental Fig. 1. The values represent the peak and tail amplitudes of the individual input functions

The manual and automated extraction of the carotid artery resulted in activity concentrations over time highly correlated with those from the continuous sampling of blood from the radial artery (Fig. 2, Supplemental Fig. 1). Delay-corrected AIF and the four corresponding IDIF showed mostly visual overlap between the curves (Fig. 2). Differences occurred mainly in the peak height and in early parts of the input functions (0–10 min post-injection). Peak and tail amplitudes of the manually and automatically segmented IDIF did not differ significantly for the most part (peak amplitudes: IDIF manual, avg vs. IDIF automated, avg: p = 0.11, IDIF manual, max5 vs. IDIF automated, max: p = 0.11; tail amplitudes: IDIF manual, avg vs. IDIF automated, avg: p = 0.76, IDIF manual, max5 vs. IDIF automated, max: p = 0.03).

**Fig. 3:**
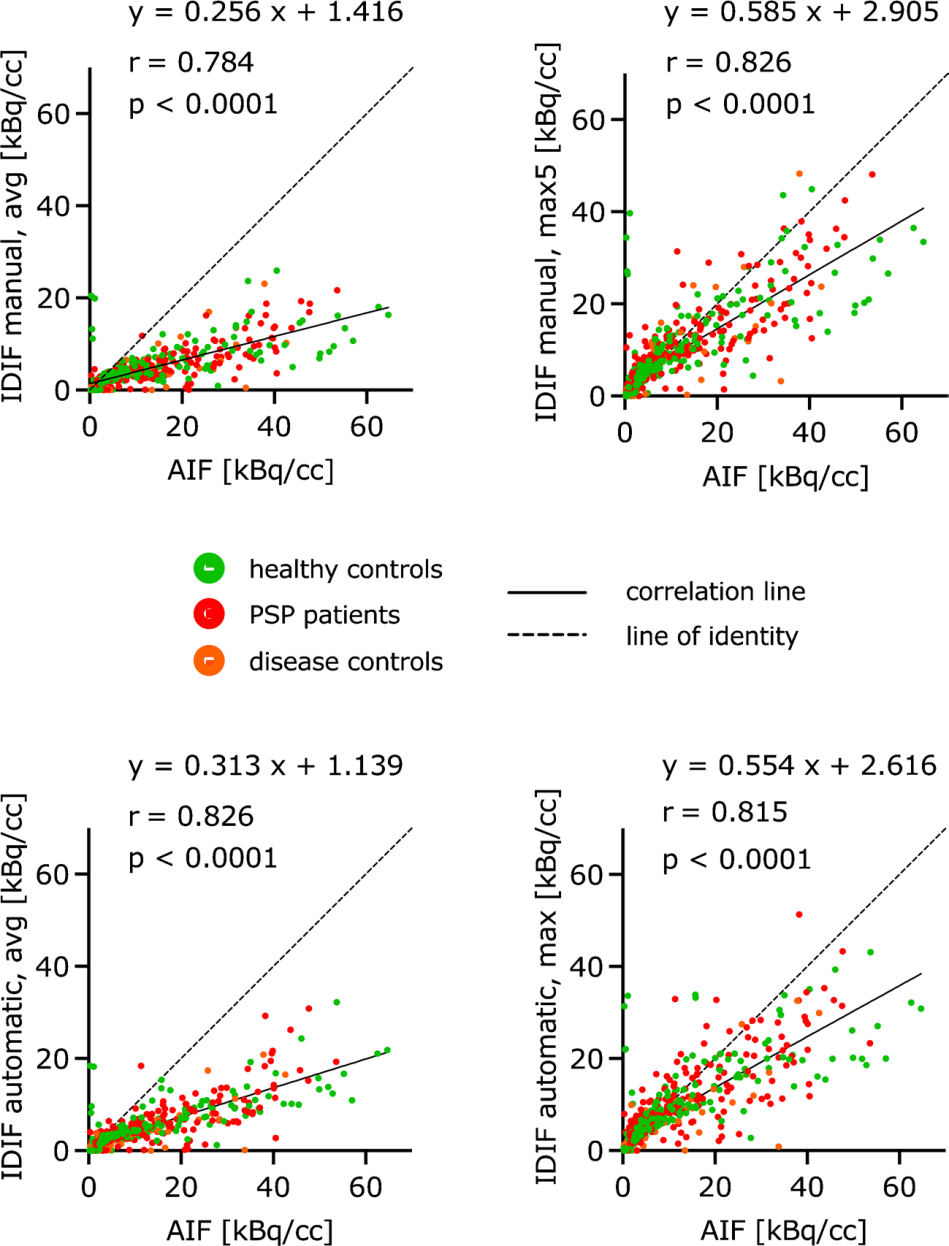
Correlations between AIF and IDIF generated by manual and automated methods (healthy controls: n = 8, PSP patients: n = 10, disease controls: n = 2)

Highest correlations of input curves and respective AUC values were observed between the AIF and the IDIF generated by the manual method using the five highest voxel intensity values (max5) (Fig. 3, Supplemental Fig. 2). However, correlation coefficients of input curves and AUC were consistently at high level for all applied IDIF methods. Methods using the maximum voxel intensity values were closer aligned to the line of identity when compared to average values (Fig. 3). Bland–Altman plots showed similar results (Supplemental Fig. 3). In those based on the IDIF methods with the maximum voxel intensity values, the scatter got smaller and less dependent on the absolutes.

### AIF vs. IDIF—impact on regional distribution volumes

Regional mean V_T_ values of the full cohort (n = 20) calculated with AIF and IDIF are presented in Table 2 as well as split into patients with PSP and controls in Supplemental Table 1. CoV were higher for V_T_ values calculated with the AIF when compared to V_T_ values calculated with the IDIF, independent of the applied IDIF method (all *p* < 0.0001).

**Table 2:** Comparison of regional mean V_T_ values [ml/ccm] with corresponding coefficients of variation (CoV) [%] calculated with AIF and IDIF generated by manual and automated methods (n = 20)

	AIF		IDIF manual, avg		IDIF manual, max5		IDIF automated, avg		IDIF automated, max	
	V_T_ (mean ± SD)	CoV	V_T_ (mean ± SD)	CoV	V_T_ (mean ± SD)	CoV	V_T_ (mean ± SD)	CoV	V_T_ (mean ± SD)	CoV
MPFC	0.44 ± 0.11	25.54	0.80 ± 0.13	16.67	0.47 ± 0.09	19.26	0.83 ± 0.14	16.57	0.56 ± 0.11	20.27
DLPFC	0.47 ± 0.12	25.70	0.85 ± 0.14	16.90	0.50 ± 0.09	18.66	0.87 ± 0.12	14.28	0.59 ± 0.11	18.98
Cerebellum	0.46 ± 0.11	24.38	0.84 ± 0.14	16.18	0.50 ± 0.09	17.86	0.86 ± 0.11	12.66	0.58 ± 0.10	17.74
Globus pallidus	0.58 ± 0.15	26.18	1.05 ± 0.21	19.58	0.61 ± 0.11	18.71	1.07 ± 0.16	14.59	0.73 ± 0.15	20.42
Globus pallidus externus	0.58 ± 0.15	26.51	1.06 ± 0.21	19.80	0.62 ± 0.12	18.92	1.08 ± 0.16	14.90	0.73 ± 0.15	20.57
Globus pallidus internus	0.58 ± 0.15	25.33	1.05 ± 0.20	19.09	0.61 ± 0.11	18.16	1.07 ± 0.15	13.71	0.72 ± 0.15	20.20
Dentate nucleus	0.56 ± 0.14	24.95	1.02 ± 0.18	17.84	0.59 ± 0.11	18.26	1.04 ± 0.14	13.25	0.70 ± 0.14	19.29
Subthalamic nucleus	0.55 ± 0.13	23.62	1.01 ± 0.17	17.07	0.59 ± 0.10	17.35	1.03 ± 0.12	11.79	0.70 ± 0.13	18.58
Putamen	0.57 ± 0.15	26.41	1.03 ± 0.19	18.66	0.60 ± 0.11	19.00	1.06 ± 0.16	15.21	0.72 ± 0.15	20.90
Substantia nigra	0.52 ± 0.14	26.53	0.93 ± 0.16	16.78	0.55 ± 0.11	19.39	0.96 ± 0.13	14.11	0.65 ± 0.13	19.71
Dorsal midbrain	0.45 ± 0.12	26.86	0.82 ± 0.17	20.30	0.48 ± 0.11	22.05	0.83 ± 0.13	15.35	0.57 ± 0.13	22.28

**Fig. 4:**
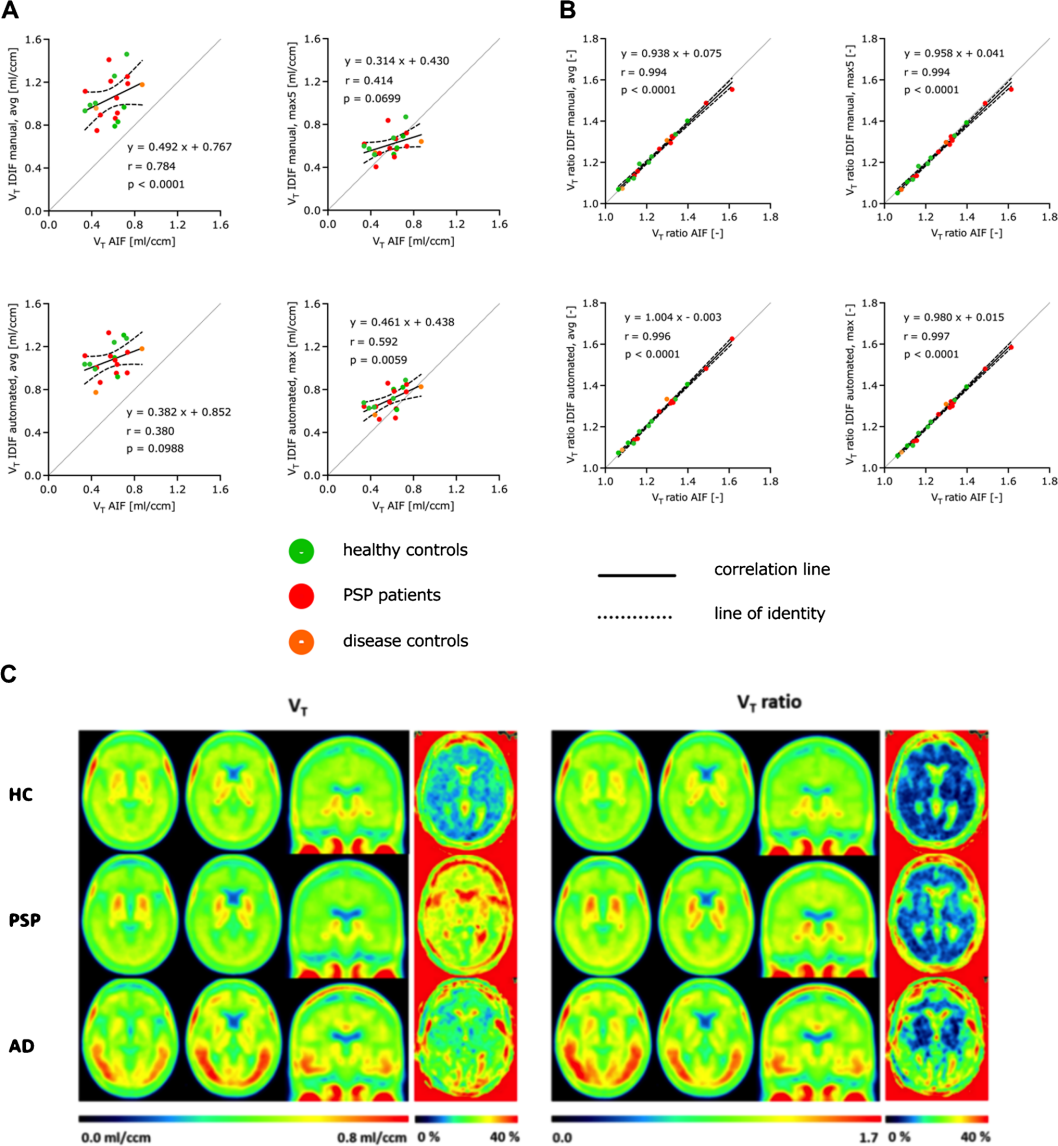
The globus pallidus internus was the most relevant PSP target region for [^18^F]PI-2620 PET imaging in previous studies ^2^ and served as an exemplary region of interest. (**A**) Correlations of V_T_ values [ml/ccm] in the globus pallidus internus calculated with AIF and IDIF generated by manual and automated methods with confidence intervals of 95% (healthy controls: n = 8, PSP patients: n = 10, disease controls: n = 2). (**B**) Correlations of V_T_ ratio values [-] (reference region: inferior cerebellum) in the globus pallidus internus calculated with AIF and IDIF generated by manual and automated methods with confidence intervals of 95% (healthy controls: n = 8, PSP patients: n = 10, disease controls: n = 2). (**C**) Averaged V_T_ [ml/ccm] and V_T_ ratio [-] images with corresponding coefficient of variation [%] image of healthy controls (HC) (n = 15), patients with PSP (n = 15) and patients with AD (n = 10)

Table 3 shows the absolute and relative differences between the regional V_T_ values calculated with AIF and the corresponding V_T_ values calculated with the manually and automatically segmented IDIF. Averaged over all regions, the V_T_ values of the manual IDIF method using the average voxel intensity values deviate -82%, those of the manual IDIF method using the five highest voxel intensity values (max5) -6%, those of the automated method using average voxel intensity values -86% and those of the automated method using the maximum voxel value -26% from the V_T_ values of the AIF. Even the Bland–Altman plots showed the smallest differences and thus the best agreement between the V_T_ calculated with AIF and with IDIF generated by the manual IDIF method using the five highest voxel intensity values (max5) (Supplemental Fig. 4). Correlations between regional V_T_ values obtained from AIF and IDIF were found in some cases, mainly for those IDIF using the maximum voxel intensity values (Fig. 4 A, Table 4).

**Table 3:** Absolute (AD) and relative (RD) differences between regional VT [ml/ccm] calculated with AIF and corresponding regional VT [ml/ccm] calculated with IDIF generated by manual and automated methods (n = 20)

	IDIF manual, avg		IDIF manual, max5		IDIF automated, avg		IDIF automated, max	
	AD [ml/ccm]	RD [%]	AD [ml/ccm]	RD [%]	AD [ml/ccm]	RD [%]	AD [ml/ccm]	RD [%]
MPFC	-0.36	-82	-0.03	-7	-0.38	-87	-0.12	-26
DLPFC	-0.38	-81	-0.03	-6	-0.40	-85	-0.12	-25
Cerebellum	-0.38	-82	-0.03	-7	-0.40	-86	-0.12	-26
Globus pallidus	-0.47	-82	-0.03	-6	-0.50	-86	-0.15	-26
Globus pallidus externus	-0.48	-82	-0.03	-6	-0.50	-86	-0.15	-26
Globus pallidus internus	-0.47	-82	-0.03	-6	-0.50	-86	-0.15	-26
Dentate nucleus	-0.46	-82	-0.04	-6	-0.48	-86	-0.14	-25
Subthalamic nucleus	-0.45	-82	-0.04	-6	-0.47	-86	-0.14	-26
Putamen	-0.46	-82	-0.03	-6	-0.49	-86	-0.15	-26
Substantia nigra	-0.42	-81	-0.03	-6	-0.44	-85	-0.13	-26
Dorsal midbrain	-0.37	-83	-0.03	-7	-0.39	-86	-0.12	-28
Overall	-0.43	-82	-0.03	-6	-0.45	-86	-0.14	-26

**Table 4:** Pearson correlation coefficients for regional mean V_T_ and V_T_ ratio values calculated with AIF and regional mean V_T_ and V_T_ ratio values calculated with IDIF generated by manual and automated methods (n = 20, * *p* < 0.05)

	V_T_				V_T_ ratio			
	IDIF manual, avg	IDIF manual, max5	IDIF auto-mated, avg	IDIF auto-mated, max	IDIF manual, avg	IDIF manual, max5	IDIF auto-mated, avg	IDIF auto-mated, max
MPFC	0.23 (ns)	0.41 (ns)	0.39 (ns)	0.50 (*)	1.00 (*)	1.00 (*)	1.00 (*)	1.00 (*)
DLPFC	0.31 (ns)	0.45 (*)	0.42 (ns)	0.53 (*)	0.99 (*)	0.92 (*)	0.91 (*)	1.00 (*)
Cerebellum	0.23 (ns)	0.34 (ns)	0.29 (ns)	0.42 (ns)	1.00 (*)	1.00 (*)	1.00 (*)	1.00 (*)
Globus pallidus	0.39 (ns)	0.45 (*)	0.42 (ns)	0.51 (*)	0.99 (*)	0.96(*)	0.97 (*)	1.00 (*)
Globus pallidus externus	0.40 (ns)	0.46 (*)	0.44 (ns)	0.52 (*)	0.99 (*)	0.96 (*)	0.97 (*)	1.00 (*)
Globus pallidus internus	0.36 (ns)	0.41 (ns)	0.38 (ns)	0.50 (ns)	0.99 (*)	0.94 (*)	0.96 (*)	1.00 (*)
Dentate nucleus	0.31 (ns)	0.43 (ns)	0.35 (ns)	0.49 (*)	0.99 (*)	1.00 (*)	1.00 (*)	1.00 (*)
Subthalamic nucleus	0.23 (ns)	0.35 (ns)	0.24 (ns)	0.40 (ns)	0.99 (*)	0.95 (*)	0.97 (*)	1.00 (*)
Putamen	0.37 (ns)	0.47 (*)	0.45 (*)	0.50 (*)	1.00 (*)	0.98 (*)	0.99 (*)	1.00 (*)
Substantia nigra	0.32 (ns)	0.47 (*)	0.42 (ns)	0.48 (*)	0.99 (*)	0.97 (*)	0.97 (*)	1.00 (*)
Dorsal midbrain	0.38 (ns)	0.49 (*)	0.39 (ns)	0.41 (ns)	1.00 (*)	1.00 (*)	1.00 (*)	1.00 (*)

Applying reference tissue scaling by the inferior cerebellar grey matter led to strong reduction of CoV at the group level and revealed nearly perfect agreement between V_T_ ratio values of AIF and IDIF (Fig. 4 B, Table 4, Supplemental Table 2, Supplemental Table 3).

### Evaluation of IDIF Based Volumes of Distribution in an Independent Cohort

After validation of IDIF against the AIF gold-standard, we evaluated the quantification of [^18^F]PI-2620 binding by estimation of regional V_T_ using IDIF in an independent cohort of 40 individuals with dynamic [^18^F]PI-2620 tau-PET imaging but without arterial sampling. To this end, IDIF generated by the manual method with the five highest voxel intensity values (max5) were used, since they showed high correlations with AIF, also in terms of the AUC, and the smallest differences in the activity concentrations and regional V_T_ compared to these of the AIF.

High standard deviations and CoV were observed for V_T_ in all groups (healthy controls, PSP, AD) in all target regions. Apart from Braak region I and V, no significant differences were obtained between the patients and healthy controls (Supplemental Table 4, upper part).

Next, we applied reference region scaling of V_T_ images to test if detection of group differences between patients with PSP or AD and healthy controls can be improved by ratio images. The inferior cerebellum was used as a reference region since it did not show differences of [^18^F]PI-2620 V_T_ between healthy controls, PSP patients and AD patients. CoV of regional V_T_ ratio values were lower compared to corresponding CoV of V_T_ values (Fig. 4 C). Significant differences in regional mean V_T_ ratio values among groups were observed in all regions except the medial prefrontal cortex, inferior cerebellum, dentate nucleus, putamen, substantia nigra and dorsal midbrain (Supplemental Table 4, lower part).

**Fig. 5:**
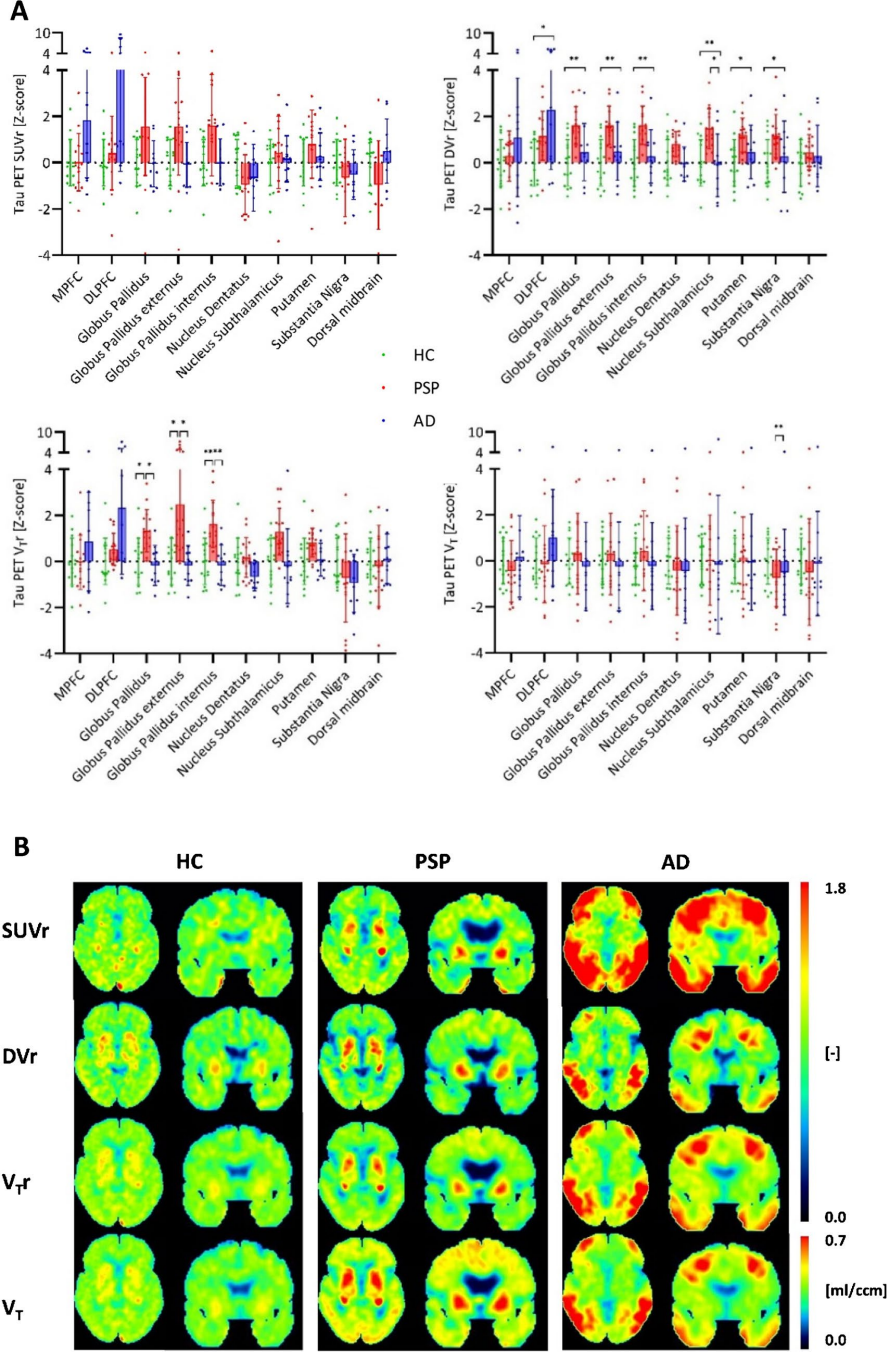
Comparison of SUV ratio (SUVr), DV ratio (DVr), V_T_ ratio (V_T_r) and V_T_ in PSP target regions. (A) SUVr, DVr, VTr and V_T_ values [Z-score] in PSP target regions (healthy controls: n = 15, PSP patients: n = 15, AD patients: n = 10); significant differences are presented as follows: * *p* < 0.05, ** *p* < 0.01, *** *p* < 0.001, **** *p* < 0.0001. (B) SUVr [-], DVr [-], V_T_r [-] and V_T_ [ml/ccm] images of basal ganglia of a healthy control (HC), a patient with PSP and a patient with AD

**Fig. 6:**
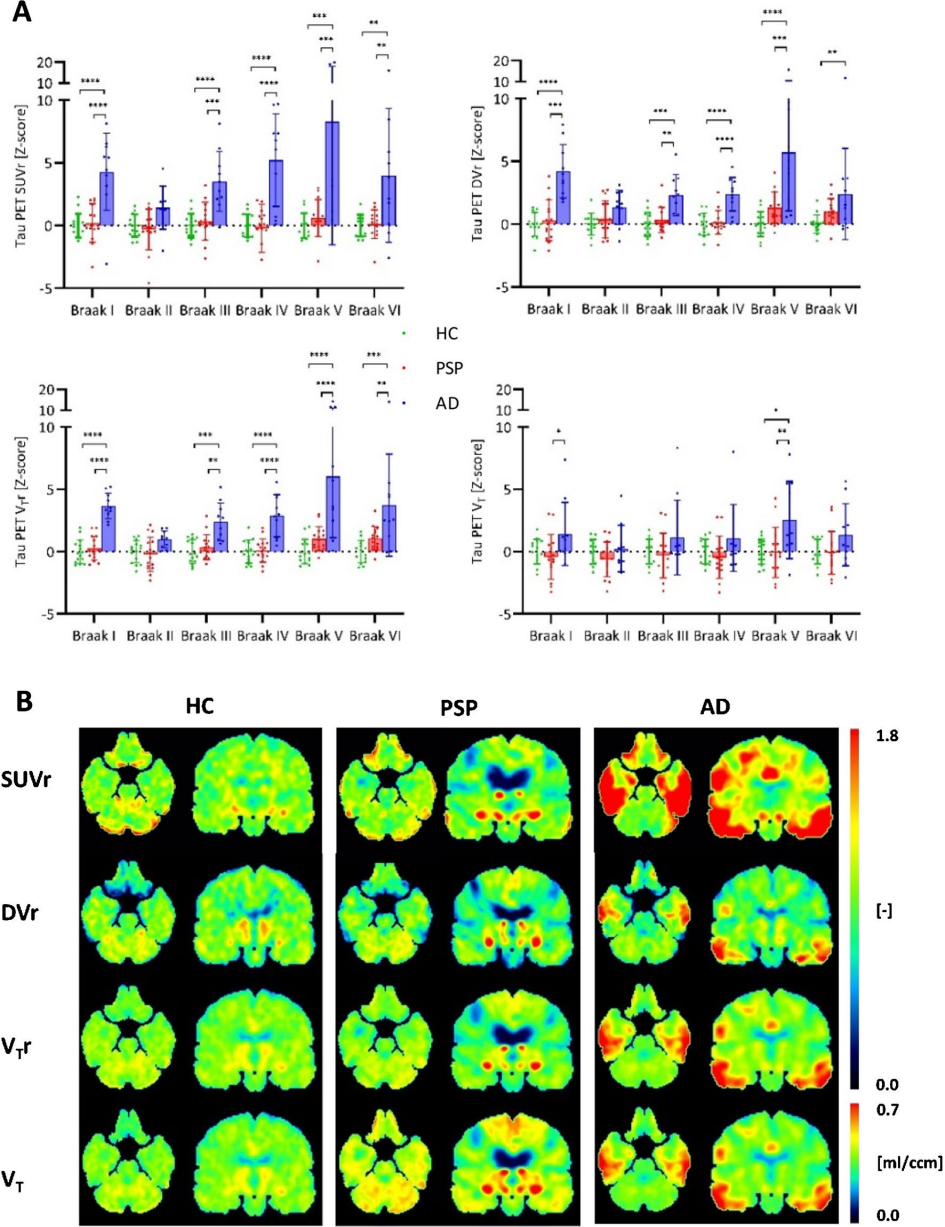
Comparison of SUV ratio (SUVr), DV ratio (DVr), V_T_ ratio (V_T_r) and V_T_ in AD target regions. (A) SUVr, DVr, V_T_r and V_T_ values [Z-score] in Braak regions (healthy controls: n = 15, PSP patients: n = 15, AD patients: n = 10); significant differences are presented as follows: * *p* < 0.05, ** *p* < 0.01, *** *p* < 0.001, **** *p* < 0.0001. (B) SUVr [-], DVr [-], V_T_r [-] and V_T_ [ml/ccm] images of hippocampus of a healthy control (HC), a patient with PSP and a patient with AD

Regarding PSP target regions, patients with PSP had highest V_T_ ratios in the globus pallidus (HC vs. PSP vs. AD: 1.18 vs. 1.32 vs. 1.16; Supplemental Table 4, lower part). For patients with AD, strongest significant V_T_ ratio differences were observed in Braak region I (HC vs. PSP vs. AD: 1.00 vs. 1.00 vs. 1.22; Supplemental Table 4, lower part). Finally, we compared V_T_ and V_T_ ratios as indices of IDIF based [^18^F]PI-2620 quantification with SUV ratios and DV ratios (Figs. 5 and 6). V_T_ were positively correlated with V_T_ ratios, DV ratios, and SUV ratios at a moderate to strong level (Supplemental Table 5). V_T_ ratios and DV ratios showed higher quantitative differences in PSP target regions between healthy controls and patients with PSP when compared to SUV ratios or V_T_ (Cohen’s D in globus pallidus: d(SUV ratio) = 0.800, d(DV ratio) = 1.562, d(V_T_ ratio) = 1.260, d(V_T_) = 0.221) (Fig. 5). In the comparison of healthy controls and patients with AD, quantitative differences in Braak I-VI regions were similar for V_T_ ratios, DV ratios and SUV ratios, whereas V_T_ resulted in lower z-scores due to larger variance at the group level (Cohen’s D in Braak I: d(SUV ratio) = 2.064, d(DV ratio) = 2.769, d(V_T_ ratio) = 3.717, d(V_T_) = 0.798) (Fig. 6).

## Discussion

### Comparison of input functions

This work validated quantification of [^18^F]PI-2620 tau-PET by use of a completely blood-free IDIF to facilitate quantification of tracer binding in brain without continuous sampling of whole blood from the radial artery. Additionally, we established assessment of the IDIF by a fully automated extraction of the PET signal from the carotid artery, which allows operator-independent [^18^F]PI-2620 quantification via IDIF in large cohorts.

AIF and the IDIF closely matched in their shapes and activity concentrations, while the main differences occurred in the peak height and early parts of the input functions, independent of the use of average or maximum voxels. Since the relative area under the peak was quite small compared to the area under the total input function, these alterations in the peak height of the IDIF might be negligible when using analysis methods based on time-integrated data. In contrast, when using other kinetic modelling methods, such as the two-tissue compartment model, the different peak magnitudes could potentially have an impact on the parameter estimates, especially on the parameter K1, which could lead to inaccuracies or bias. Differences in early parts of the input functions (0–10 min post-injection) could be explained by the fact that the continuous blood measurements are taken every second, while the IDIF represented an average over each PET frame duration [25]. Furthermore, the AIF could be affected by several issues related to the blood sampling procedure (e.g. pumping speed, tube length) while the accuracy of the IDIF depends on partial volume effect, including spill-in and spill-out effects as well as image noise [8, 25, 26]. It should also be mentioned at this point that, unlike analysis methods relying on image data only, externally measured input functions depend on precise scanner calibration.

While our study aimed at generating input functions without the need of any blood sample, our validation data could also be used for testing the effect of IDIF calibration using arterial samples. In a subset of data from part I of the study we have used late (30–60 min post-injection) AIF values for calibrating the IDIF generated by the manual method using the five highest voxel intensity values (max5). Resulting AUC values differed on average by 27%, a value similar to the high CoV in V_T_. Thus, calibration of the IDIF generated by the manual method using the five highest voxel intensity values (max5) seems to have little effect, as reported previously by Mourik et al. [27] for a different tracer. Further evaluation of different calibration and spill-over correction methods will be the focus of a separate study.

Overall, there was good agreement between the AIF and the IDIF using maximum voxel intensity values and also between the AUC of the respective input functions, which shows that the IDIF methods worked robustly to quantify the radioactivity concentrations in arterial whole blood. Since the difference was small, manual and automated extraction of the PET signal from carotid artery can be used as surrogates for AIF. In terms of practicability, the automated IDIF extraction has a strong advantage with regard to workload reduction and operator independence.

### [^18^F]PI-2620 quantification

For quantification of [^18^F]PI-2620 uptake, regional V_T_ values were calculated by use of AIF and IDIF for calculation of Logan plots. Correlations were observed mainly between regional V_T_ values calculated with AIF and with IDIF using the maximum voxel intensity values. Also, when comparing the relative differences between V_T_ calculated with AIF and IDIF, those generated by the IDIF methods using the maximum voxel intensity values showed much lower deviations (below 26%) than the V_T_ generated by the IDIF methods using average voxel intensity values.

When considering V_T_ values of the PSP target regions examined in the cohort consisting of 20 subjects with invasive sampling, coefficients of variation of up to 27% were observed, ranging even higher for AIF compared to IDIF. Variance in V_T_ might arise from methodological obstacles such as dispersion (AIF), cardiac output (AIF and IDIF) or partial volume effects as well as from physiological variance in off-target binding and age-related target binding [28]. Therefore, we explored the potential value of V_T_ ratios. The inferior cerebellum served as reference region, since previous studies have shown low overall V_T_ and variability in V_T_ as well as no significant tau deposition in inferior cerebellum [4, 29]. As expected, coefficients of variation of V_T_ ratios were lower than those of V_T_ and differences between patients with PSP and healthy controls aligned in topology with our earlier data using DV ratios [2], as well as with the DV ratios obtained in this study.

Nevertheless, it could be useful to perform IDIF based [^18^F]PI-2620 V_T_ group comparisons with larger cohorts to investigate whether the variance can be reduced and thus reference region scaling of V_T_ can be avoided. It would allow regional differences in tracer binding to be detected without potential bias by signal changes in the reference tissue. This could be highly relevant for 4R tauopathies since cerebellar tau deposition can occur in PSP according to histopathological studies of tau spreading during the disease course [8]. Furthermore, predilection of cerebellar tau deposition can also occur in rare PSP phenotypes [30]. Thus, comparison of cerebellar [^18^F]PI-2620 V_T_ between patients with PSP and healthy controls may shed additional light on changes of the hitherto applied reference tissue during the disease course. Although cerebellar tau deposition is rare in AD, early onset phenotypes can also be associated with increased p-tau in the cerebellum [31]. In this regard, calculation of V_T_ allows to compare quantification of tracer binding without bias by altered cerebral blood flow [32], which is in contrast to the use of late phase SUV. This method may help in selecting an appropriate reference tissue when regional changes in tracer delivery could occur in longitudinal studies on patients with 4R tauopathies.

### Limitations

Among the limitations of our study, it has to be considered that the AIF were not corrected for dispersion effects nor were the IDIF corrected for partial volume effects. This might affect the shapes and activity concentrations of the input functions, which have a substantial influence on Logan plots. With regard to partial volume effects correction, however, it should be noted that image corrections depend on many parameters and are error-prone. Instead, it has been shown that calibrating the IDIF with blood samples is a valid method to recover the true input function. Here, our goal was to establish a blood-free IDIF method [8, 29].

Furthermore, we considered whole blood for both AIF and IDIF, but analyses regarding plasma to whole blood ratio and radiometabolites need to be performed to assess whether the use of uncorrected whole blood (both in PET image as well as arterial sampling) is acceptable. An ongoing study is focusing on this question and we have not found any major group differences in [^18^F]PI-2620 radiometabolite concentration so far (Supplemental Fig. 5). Thus, a population-based radiometabolite correction for IDIF might be appropriate.

Also, it might be useful to analyse even larger cohorts to further evaluate if discrimination between patients with PSP or AD and healthy controls by V_T_ is possible. It should be taken into account that there are very small structures, such as the globus pallidus internus, whose visualization may be limited by the spatial resolution of PET. Absolute quantification values of such regions thus require critical consideration in particular.

## Conclusion

This study shows promise that IDIF can facilitate quantification of diagnostic PET tracer [^18^F]PI-2620 binding in brain, negating invasive arterial blood sampling. Regarding all comparative parameters, the manual IDIF method using the five highest voxel intensity values (max5) showed the best results. In order to differentiate patients with PSP and AD and healthy controls, V_T_ is not sufficient for our cohort sizes, but V_T_ ratios or DV ratios using the inferior cerebellum as reference region are required. Additional studies need to focus on larger cohorts, radiometabolite analysis as well as plasma to whole blood ratios.

### Supplementary Information

Below is the link to the electronic supplementary material.

Supplementary file1 (DOCX 1715 KB)

## Data Availability

The datasets generated during and/or analysed during the current study are available from the corresponding author on reasonable request.
