# Supplementary material for: Assessment of [18F]PI-2620 Tau-PET Quantification via Non-Invasive Automatized Image Derived Input Function
Source: Eur J Nucl Med Mol Imaging. 2024 May 8;51(11):3252–66. doi: 10.1007/s00259-024-06741-7 (PMC11368995; doi:10.1007/s00259-024-06741-7)
Supplement: Supplementary file 1 — Supplementary file1 (DOCX 1715 KB) [file 259_2024_6741_MOESM1_ESM.docx]

**SUPPLEMENT**


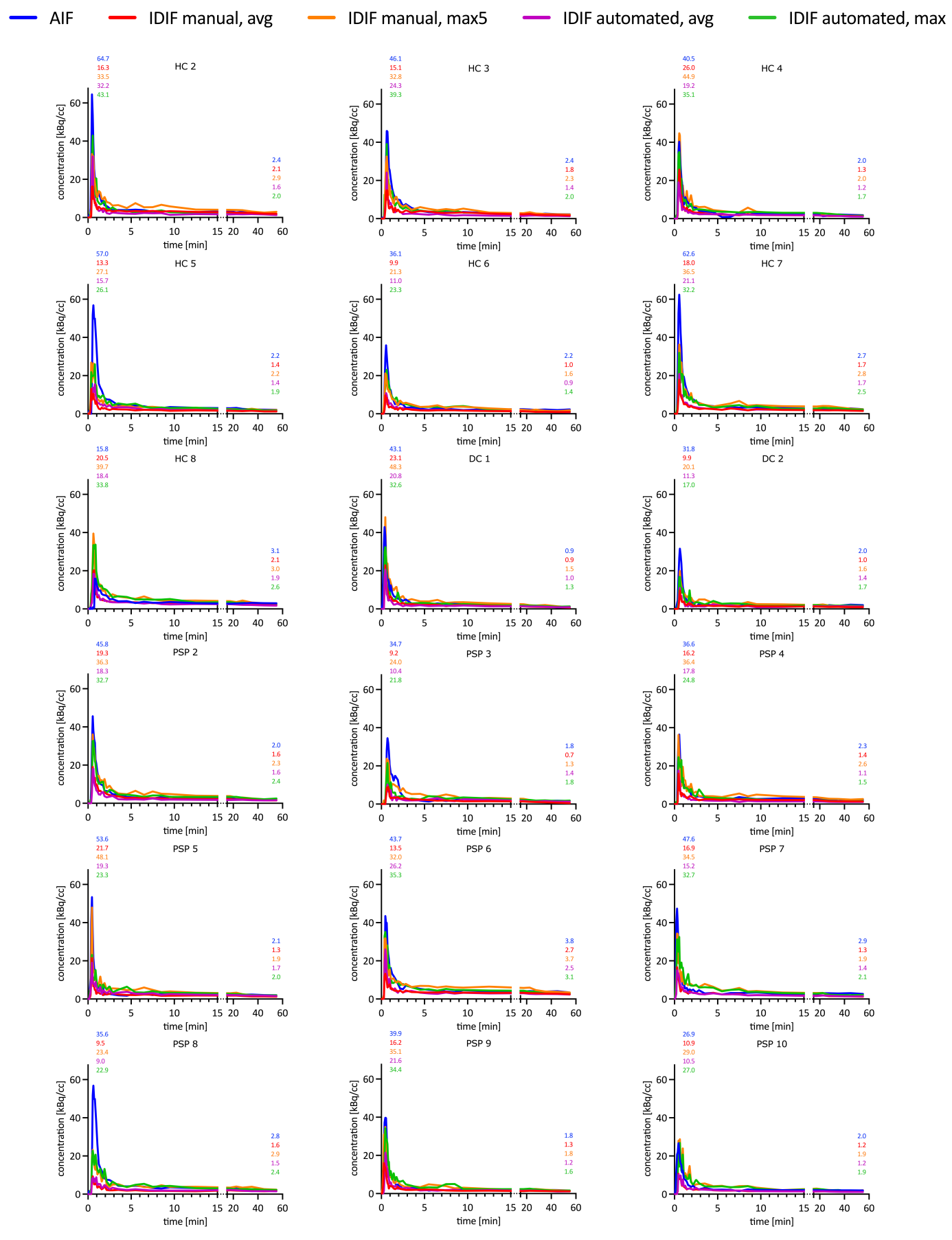


Supplemental Figure 1: Comparison of AIF and IDIF generated with the manual and automated methods on the further healthy controls and PSP patients and on the disease controls (DC 1: Parkinson disease (PD), DC 2: Frontotemporal dementia (FTD)). The values represent the peak and tail amplitudes of the individual input functions.


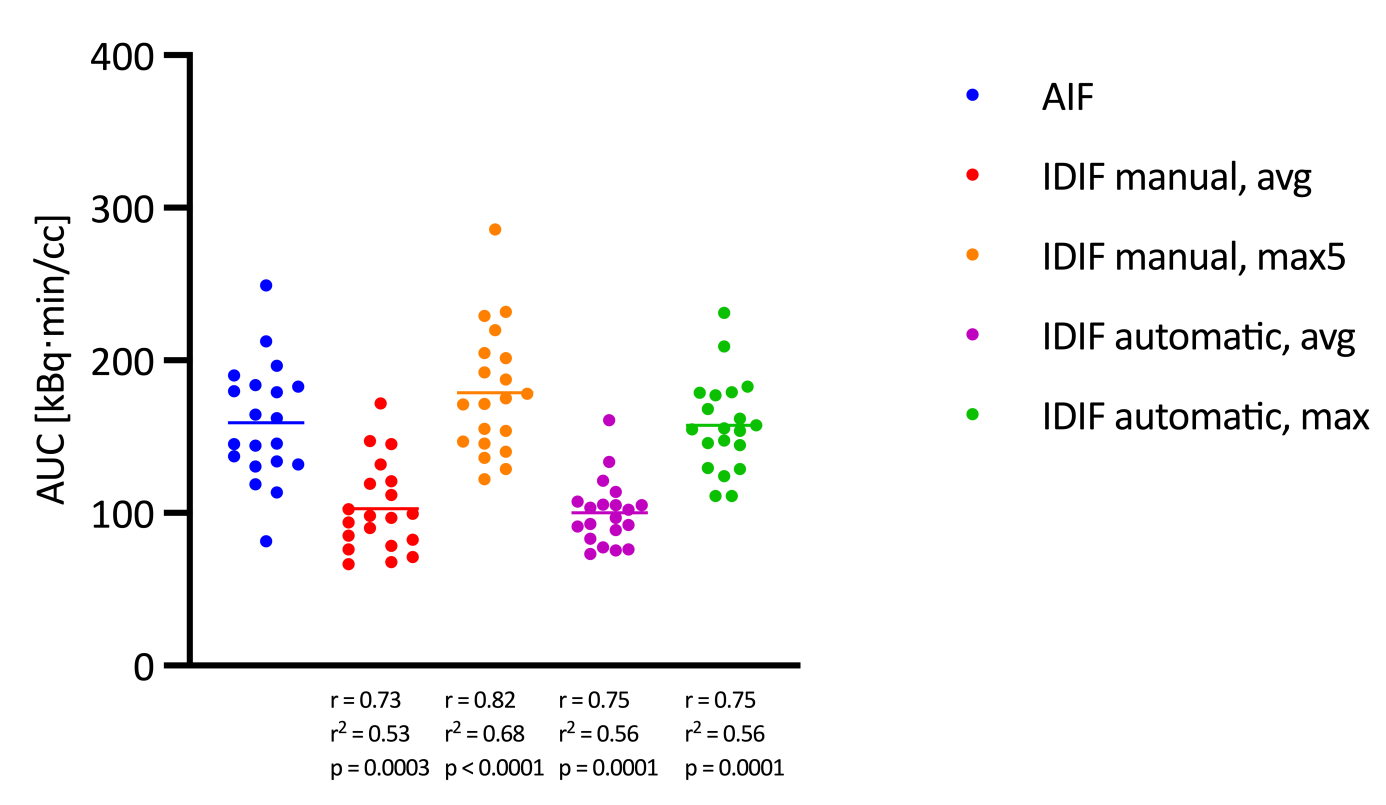


Supplemental Figure 2: Area under the curves [kBq∙min/cc] of AIF and IDIF generated by manual and automated methods (n = 20). Repeated measures ANOVA: F = 95.40, p < 0.0001.


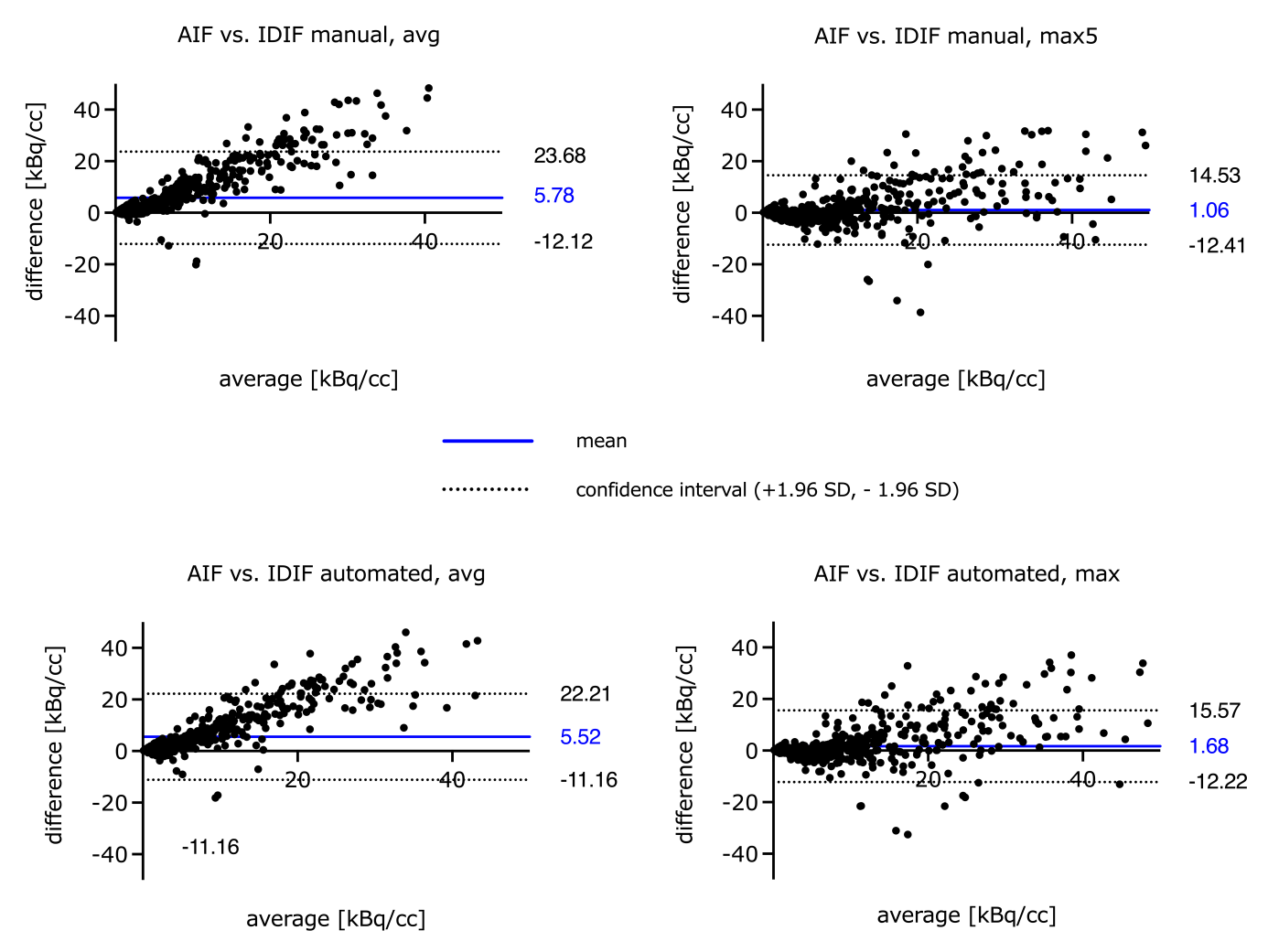


Supplemental Figure 3: Bland-Altman plot describing the differences between the activity concentrations of the AIF and one of IDIF against the mean of the respective two activity concentrations (n = 20)


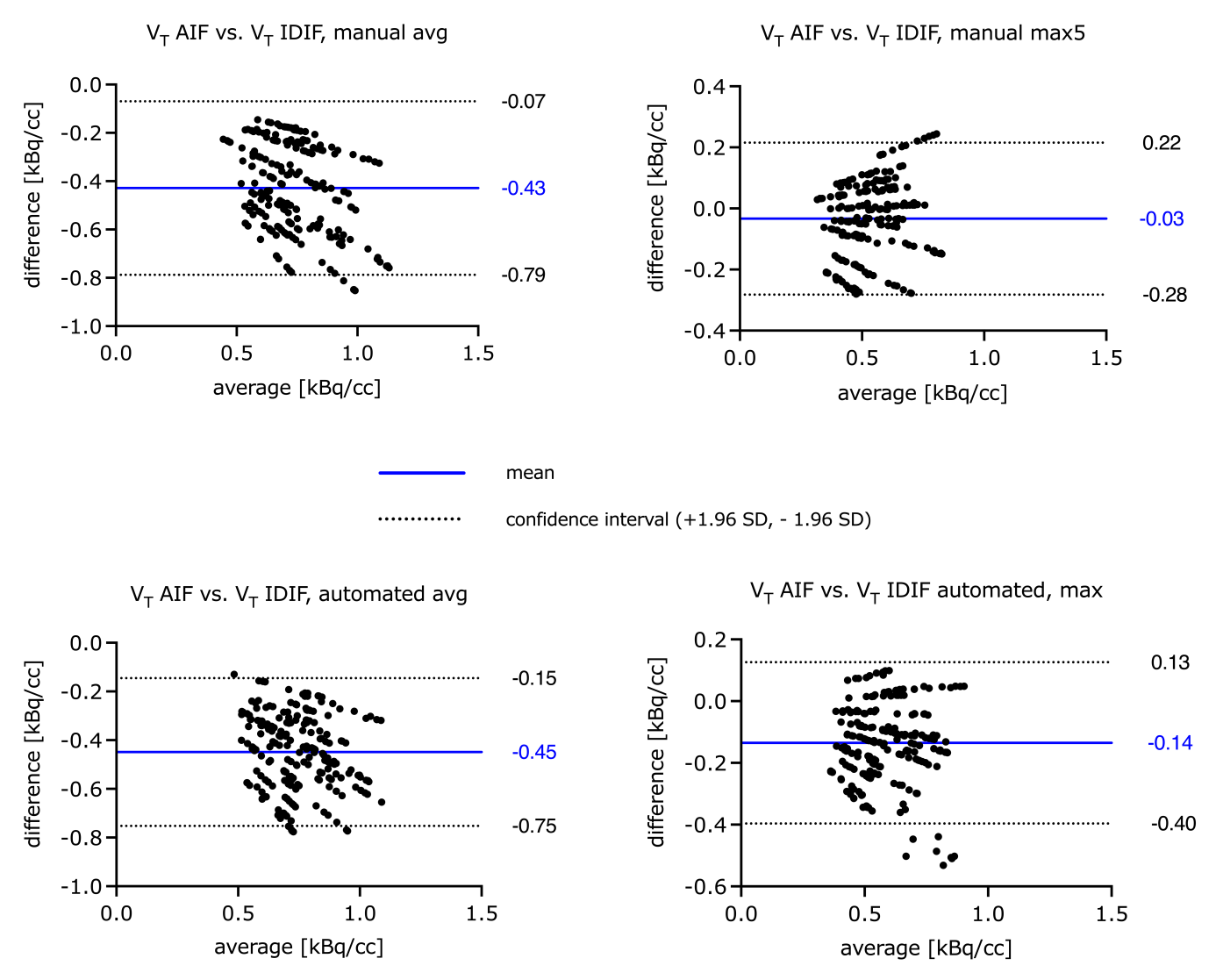


Supplemental Figure 4: Bland-Altman plot describing the differences between the regional V_T_ values calculated with AIF and one of the IDIF generated by manual and automated methods against the mean of the respective two regional V_T_ values (n = 20)


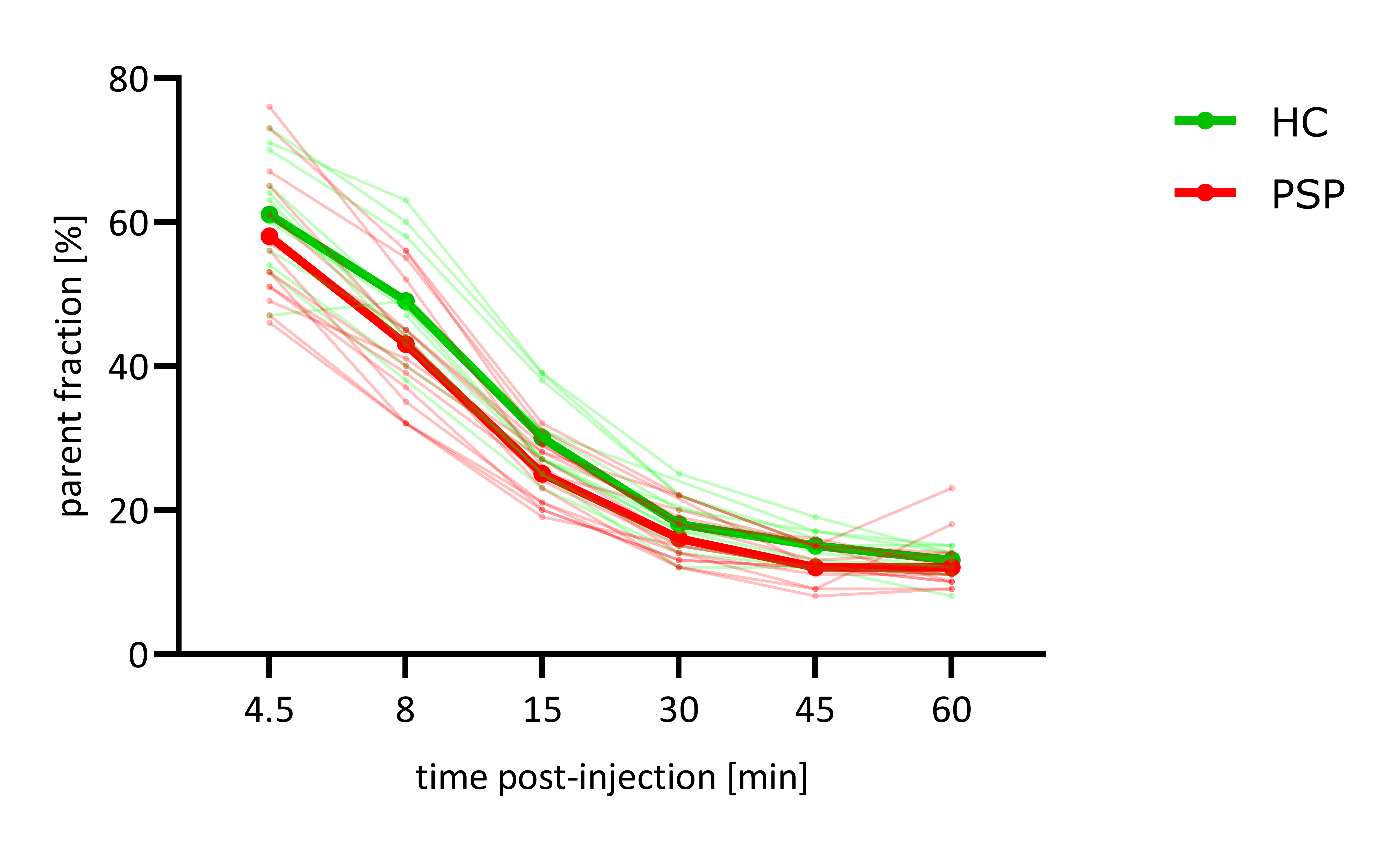


Supplemental Figure 5: Parent fraction of PSP patients and healthy controls (HC) at time points 4.5, 8, 15, 30, 56, and 60 minutes after injection. The thick lines in the front represent the mean value of the respective group. Determination based on arterial blood samples. A multiple t-test showed no significant differences between the parent fraction of the PSP patients (n = 16) and that of the healthy controls (n = 12).

|  | | **AIF** | | **IDIF manual, avg** | | **IDIF manual, max5** | | **IDIF automated, avg** | | **IDIF automated, max** | |
| --- | --- | --- | --- | --- | --- | --- | --- | --- | --- | --- | --- |
|  |  | V_T_ (mean±SD) | CoV | V_T_ (mean±SD) | CoV | V_T_ (mean±SD) | CoV | V_T_ (mean±SD) | CoV | V_T_ (mean±SD) | CoV |
| **PSP patients** | **MPFC** | 0.41 ± 0.08 | 19.47 | 0.76 ± 0.13 | 16.81 | 0.43 ± 0.09 | 20.14 | 0.76 ± 0.12 | 15.77 | 0.49 ± 0.08 | 15.40 |
|  | **DLPFC** | 0.45 ± 0.10 | 21.64 | 0.83 ± 0.14 | 16.70 | 0.47 ± 0.09 | 18.58 | 0.82 ± 0.09 | 11.20 | 0.54 ± 0.08 | 15.07 |
|  | **Cerebellum** | 0.44 ± 0.09 | 19.81 | 0.82 ± 0.13 | 16.13 | 0.47 ± 0.09 | 19.49 | 0.81 ± 0.08 | 10.43 | 0.53 ± 0.06 | 11.82 |
|  | **Globus pallidus** | 0.57 ± 0.12 | 21.55 | 1.06 ± 0.21 | 19.86 | 0.60 ± 0.12 | 20.08 | 1.05 ± 0.13 | 12.75 | 0.69 ± 0.12 | 17.08 |
|  | **Globus pallidus externus** | 0.58 ± 0.12 | 21.67 | 1.06 ± 0.21 | 20.00 | 0.60 ± 0.12 | 20.15 | 1.06 ± 0.14 | 12.90 | 0.69 ± 0.12 | 16.99 |
|  | **Globus pallidus internus** | 0.58 ± 0.13 | 21.72 | 1.07 ± 0.21 | 19.40 | 0.60 ± 0.12 | 20.00 | 1.06 ± 0.13 | 12.18 | 0.69 ± 0.12 | 17.53 |
|  | **Dentate nucleus** | 0.53 ± 0.12 | 22.23 | 0.98 ± 0.19 | 19.34 | 0.56 ± 0.12 | 21.06 | 0.97 ± 0.11 | 11.35 | 0.64 ± 0.10 | 16.05 |
|  | **Subthalamic nucleus** | 0.54 ± 0.11 | 21.07 | 0.99 ± 0.18 | 17.69 | 0.56 ± 0.11 | 19.44 | 0.99 ± 0.11 | 10.97 | 0.65 ± 0.10 | 15.80 |
|  | **Putamen** | 0.55 ± 0.11 | 20.88 | 1.02 ± 0.20 | 19.33 | 0.58 ± 0.11 | 19.73 | 1.01 ± 0.13 | 12.92 | 0.66 ± 0.11 | 17.04 |
|  | **Substantia nigra** | 0.49 ± 0.10 | 19.98 | 0.90 ± 0.16 | 17.47 | 0.51 ± 0.10 | 20.00 | 0.90 ± 0.10 | 11.68 | 0.59 ± 0.09 | 15.73 |
|  | **Dorsal midbrain** | 0.42 ± 0.07 | 17.35 | 0.79 ± 0.16 | 19.90 | 0.45 ± 0.10 | 22.97 | 0.78 ± 0.11 | 14.64 | 0.51 ± 0.08 | 16.03 |
| **Healthy and disease controls** | **MPFC** | 0.48 ± 0.13 | 28.26 | 0.85 ± 0.13 | 15.17 | 0.52 ± 0.08 | 15.08 | 0.89 ± 0.12 | 13.82 | 0.62 ± 0.11 | 17.92 |
|  | **DLPFC** | 0.49 ± 0.14 | 29.06 | 0.88 ± 0.15 | 17.31 | 0.53 ± 0.09 | 17.46 | 0.92 ± 0.14 | 14.98 | 0.64 ± 0.12 | 18.52 |
|  | **Cerebellum** | 0.48 ± 0.13 | 27.78 | 0.87 ± 0.14 | 16.39 | 0.53 ± 0.08 | 15.15 | 0.91 ± 0.11 | 12.24 | 0.64 ± 0.11 | 17.57 |
|  | **Globus pallidus** | 0.58 ± 0.18 | 31.17 | 1.05 ± 0.21 | 20.35 | 0.63 ± 0.11 | 18.09 | 1.10 ± 0.18 | 16.55 | 0.76 ± 0.17 | 22.36 |
|  | **Globus pallidus externus** | 0.59 ± 0.19 | 31.64 | 1.05 ± 0.22 | 20.67 | 0.63 ± 0.12 | 18.44 | 1.10 ± 0.19 | 16.97 | 0.77 ± 0.17 | 22.60 |
|  | **Globus pallidus internus** | 0.58 ± 0.17 | 29.72 | 1.04 ± 0.20 | 19.74 | 0.62 ± 0.11 | 17.15 | 1.09 ± 0.17 | 15.58 | 0.76 ± 0.17 | 22.14 |
|  | **Dentate nucleus** | 0.59 ± 0.16 | 27.20 | 1.05 ± 0.18 | 16.67 | 0.63 ± 0.09 | 14.16 | 1.10 ± 0.14 | 12.31 | 0.77 ± 0.14 | 17.90 |
|  | **Subthalamic nucleus** | 0.57 ± 0.15 | 26.57 | 1.02 ± 0.18 | 17.30 | 0.61 ± 0.09 | 15.22 | 1.06 ± 0.13 | 11.89 | 0.75 ± 0.14 | 18.60 |
|  | **Putamen** | 0.59 ± 0.18 | 31.29 | 1.05 ± 0.20 | 18.92 | 0.63 ± 0.11 | 18.22 | 1.10 ± 0.18 | 16.60 | 0.77 ± 0.17 | 21.58 |
|  | **Substantia nigra** | 0.54 ± 0.17 | 30.98 | 0.96 ± 0.16 | 16.29 | 0.59 ± 0.10 | 17.51 | 1.01 ± 0.14 | 14.08 | 0.71 ± 0.13 | 18.52 |
|  | **Dorsal midbrain** | 0.48 ± 0.15 | 32.15 | 0.85 ± 0.18 | 20.81 | 0.51 ± 0.10 | 20.20 | 0.89 ± 0.12 | 13.80 | 0.63 ± 0.14 | 21.70 |

Supplemental Table 1: Comparison of regional mean V_T_ values [ml/ccm] with corresponding coefficients of variation (CoV) [%] calculated with AIF and IDIF generated by manual and automated methods (healthy controls: n = 8, PSP patients: n = 10, disease controls: n = 2)

|  | **AIF** | | **IDIF manual, avg** | | **IDIF manual, max5** | | **IDIF automated, avg** | | **IDIF automated, max** | |
| --- | --- | --- | --- | --- | --- | --- | --- | --- | --- | --- |
|  | V_T_ ratio (mean±SD) | CoV | V_T_ ratio (mean±SD) | CoV | V_T_ ratio (mean±SD) | CoV | V_T_ ratio (mean±SD) | CoV | V_T_ ratio (mean±SD) | CoV |
| **MPFC** | 0.97 ± 0.07 | 7.70 | 0.97 ± 0.07 | 7.71 | 0.97 ± 0.08 | 7.82 | 0.97 ± 0.08 | 7.75 | 0.97 ± 0.08 | 7.80 |
| **DLPFC** | 1.03 ± 0.08 | 7.68 | 1.03 ± 0.07 | 7.10 | 1.02 ± 0.07 | 7.16 | 1.05 ± 0.08 | 7.78 | 1.05 ± 0.08 | 7.52 |
| **Cerebellum** | 1.01 ± 0.02 | 2.25 | 1.01 ± 0.02 | 2.29 | 1.01 ± 0.02 | 2.36 | 1.02 ± 0.02 | 2.26 | 1.02 ± 0.02 | 2.29 |
| **Globus pallidus** | 1.27 ± 0.14 | 10.83 | 1.26 ± 0.13 | 10.41 | 1.25 ± 0.13 | 10.61 | 1.29 ± 0.14 | 10.97 | 1.27 ± 0.14 | 10.83 |
| **Globus pallidus externus** | 1.27 ± 0.14 | 11.02 | 1.27 ± 0.13 | 10.62 | 1.26 ± 0.14 | 10.80 | 1.29 ± 0.14 | 11.17 | 1.28 ± 0.14 | 11.03 |
| **Globus pallidus internus** | 1.27 ± 0.14 | 11.04 | 1.26 ± 0.13 | 10.45 | 1.25 ± 0.13 | 10.74 | 1.29 ± 0.14 | 11.11 | 1.27 ± 0.14 | 10.95 |
| **Dentate nucleus** | 1.22 ± 0.08 | 6.55 | 1.22 ± 0.08 | 6.58 | 1.21 ± 0.08 | 6.88 | 1.24 ± 0.08 | 6.67 | 1.24 ± 0.08 | 6.96 |
| **Subthalamic nucleus** | 1.21 ± 0.09 | 7.05 | 1.21 ± 0.08 | 6.71 | 1.20 ± 0.08 | 6.97 | 1.24 ± 0.09 | 7.18 | 1.23 ± 0.09 | 7.16 |
| **Putamen** | 1.24 ± 0.12 | 9.93 | 1.24 ± 0.12 | 9.77 | 1.23 ± 0.12 | 10.00 | 1.27 ± 0.13 | 10.15 | 1.26 ± 0.13 | 10.21 |
| **Substantia nigra** | 1.13 ± 0.08 | 7.49 | 1.12 ± 0.08 | 7.34 | 1.12 ± 0.08 | 7.34 | 1.16 ± 0.09 | 7.69 | 1.16 ± 0.08 | 7.54 |
| **Dorsal midbrain** | 0.98 ± 0.07 | 7.64 | 0.98 ± 0.08 | 7.69 | 0.98 ± 0.07 | 7.67 | 0.98 ± 0.08 | 7.66 | 0.98 ± 0.07 | 7.65 |

Supplemental Table 2: Comparison of regional mean V_T_ ratio values [-] with corresponding coefficients of variation (CoV) [%] calculated with AIF and IDIF generated by manual and automated methods (n = 20)

|  | | **AIF** | | **IDIF manual, avg** | | **IDIF manual, max5** | | **IDIF automated, avg** | | **IDIF automated, max** | |
| --- | --- | --- | --- | --- | --- | --- | --- | --- | --- | --- | --- |
|  |  | V_T_ ratio (mean±SD) | CoV | V_T_ ratio (mean±SD) | CoV | V_T_ ratio (mean±SD) | CoV | V_T_ ratio (mean±SD) | CoV | V_T_ ratio (mean±SD) | CoV |
| **PSP patients** | **MPFC** | 0.94 ± 0.07 | 7.27 | 0.94 ± 0.07 | 7.31 | 0.94 ± 0.07 | 7.39 | 0.94 ± 0.07 | 7.56 | 0.94 ± 0.07 | 7.45 |
|  | **DLPFC** | 1.03 ± 0.08 | 7.89 | 1.03 ± 0.07 | 6.65 | 1.02 ± 0.07 | 6.67 | 1.03 ± 0.08 | 8.16 | 1.02 ± 0.08 | 7.47 |
|  | **Cerebellum** | 1.01 ± 0.03 | 2.65 | 1.01 ± 0.03 | 2.69 | 1.01 ± 0.03 | 2.83 | 1.01 ± 0.03 | 2.69 | 1.01 ± 0.03 | 2.70 |
|  | **Globus pallidus** | 1.32 ± 0.14 | 10.28 | 1.31 ± 0.12 | 9.52 | 1.30 ± 0.13 | 9.83 | 1.32 ± 0.14 | 10.58 | 1.30 ± 0.13 | 10.24 |
|  | **Globus pallidus externus** | 1.33 ± 0.14 | 10.34 | 1.32 ± 0.13 | 9.59 | 1.31 ± 0.13 | 9.88 | 1.32 ± 0.14 | 10.65 | 1.31 ± 0.14 | 10.30 |
|  | **Globus pallidus internus** | 1.33 ± 0.14 | 10.56 | 1.32 ± 0.13 | 9.68 | 1.31 ± 0.13 | 10.14 | 1.33 ± 0.14 | 10.87 | 1.31 ± 0.14 | 10.54 |
|  | **Dentate nucleus** | 1.22 ± 0.09 | 7.51 | 1.22 ± 0.09 | 7.55 | 1.21 ± 0.10 | 7.98 | 1.22 ± 0.09 | 7.62 | 1.21 ± 0.10 | 8.06 |
|  | **Subthalamic nucleus** | 1.24 ± 0.10 | 8.36 | 1.23 ± 0.10 | 7.81 | 1.23 ± 0.10 | 8.15 | 1.24 ± 0.11 | 8.54 | 1.23 ± 0.10 | 8.52 |
|  | **Putamen** | 1.27 ± 0.12 | 9.51 | 1.26 ± 0.12 | 9.14 | 1.25 ± 0.12 | 9.56 | 1.27 ± 0.13 | 9.91 | 1.25 ± 0.12 | 9.89 |
|  | **Substantia nigra** | 1.13 ± 0.10 | 8.72 | 1.12 ± 0.09 | 8.40 | 1.12 ± 0.09 | 8.45 | 1.13 ± 0.10 | 9.02 | 1.12 ± 0.10 | 8.84 |
|  | **Dorsal midbrain** | 0.97 ± 0.08 | 8.38 | 0.97 ± 0.08 | 8.33 | 0.97 ± 0.08 | 8.34 | 0.97 ± 0.08 | 8.19 | 0.97 ± 0.08 | 8.31 |
| **Healthy and disease controls** | **MPFC** | 0.99 ± 0.07 | 7.54 | 0.99 ± 0.07 | 7.38 | 0.99 ± 0.07 | 7.46 | 0.99 ± 0.07 | 7.36 | 0.99 ± 0.07 | 7.48 |
|  | **DLPFC** | 1.03 ± 0.08 | 7.87 | 1.02 ± 0.08 | 7.89 | 1.02 ± 0.08 | 7.99 | 1.03 ± 0.08 | 7.79 | 1.02 ± 0.08 | 7.96 |
|  | **Cerebellum** | 1.01 ± 0.02 | 1.90 | 1.01 ± 0.02 | 1.96 | 1.01 ± 0.02 | 1.95 | 1.01 ± 0.02 | 1.88 | 1.01 ± 0.02 | 1.93 |
|  | **Globus pallidus** | 1.21 ± 0.12 | 10.12 | 1.21 ± 0.13 | 10.33 | 1.21 ± 0.13 | 10.42 | 1.22 ± 0.13 | 10.33 | 1.21 ± 0.13 | 10.44 |
|  | **Globus pallidus externus** | 1.22 ± 0.13 | 10.60 | 1.22 ± 0.13 | 10.79 | 1.21 ± 0.13 | 10.87 | 1.23 ± 0.13 | 10.79 | 1.21 ± 0.13 | 10.91 |
|  | **Globus pallidus internus** | 1.20 ± 0.11 | 9.38 | 1.20 ± 0.11 | 9.49 | 1.20 ± 0.12 | 9.63 | 1.21 ± 0.12 | 9.55 | 1.20 ± 0.12 | 9.62 |
|  | **Dentate nucleus** | 1.23 ± 0.07 | 5.84 | 1.23 ± 0.07 | 5.84 | 1.22 ± 0.07 | 6.01 | 1.23 ± 0.07 | 5.95 | 1.22 ± 0.07 | 6.10 |
|  | **Subthalamic nucleus** | 1.19 ± 0.06 | 4.71 | 1.19 ± 0.06 | 4.88 | 1.18 ± 0.06 | 5.02 | 1.19 ± 0.06 | 4.87 | 1.18 ± 0.06 | 5.00 |
|  | **Putamen** | 1.22 ± 0.13 | 10.44 | 1.22 ± 0.13 | 10.57 | 1.21 ± 0.13 | 10.69 | 1.22 ± 0.13 | 10.61 | 1.21 ± 0.13 | 10.78 |
|  | **Substantia nigra** | 1.13 ± 0.07 | 6.51 | 1.13 ± 0.07 | 6.58 | 1.12 ± 0.07 | 6.51 | 1.13 ± 0.07 | 6.59 | 1.13 ± 0.07 | 6.48 |
|  | **Dorsal midbrain** | 0.99 ± 0.07 | 7.22 | 0.99 ± 0.07 | 7.38 | 0.98 ± 0.07 | 7.33 | 0.99 ± 0.07 | 7.43 | 0.98 ± 0.07 | 7.31 |

Supplemental Table 3: Comparison of regional mean V_T_ ratio values [-] with corresponding coefficients of variation (CoV) [%] calculated with AIF and IDIF generated by manual and automated methods (healthy controls: n = 8, PSP patients: n = 10, disease controls: n = 2)

|  |  | **Healthy controls** | | **PSP patients** | | **AD patients** | |
| --- | --- | --- | --- | --- | --- | --- | --- |
|  |  | **V_T_ (mean±SD)** | **CoV** | **V_T_ (mean±SD)** | **CoV** | **V_T_ (mean±SD)** | **CoV** |
| **PSP target regions** |  | | | | | | |
| MPFC | ns | 0.46 ± 0.08 | 16.66 | 0.42 ± 0.09 | 22.75 | 0.49 ± 0.13 | 25.74 |
| DLPFC | ns | 0.48 ± 0.07 | 14.85 | 0.46 ± 0.12 | 25.91 | 0.58 ± 0.13 | 23.06 |
| Cerebellum | ns | 0.48 ± 0.06 | 11.71 | 0.46 ± 0.11 | 23.28 | 0.48 ± 0.12 | 24.59 |
| Globus pallidus | ns | 0.55 ± 0.08 | 15.02 | 0.57 ± 0.15 | 26.42 | 0.55 ± 0.15 | 27.93 |
| Globus pallidus externus | ns | 0.55 ± 0.08 | 15.09 | 0.58 ± 0.15 | 26.76 | 0.56 ± 0.16 | 28.10 |
| Globus pallidus internus | ns | 0.54 ± 0.08 | 14.90 | 0.58 ± 0.15 | 25.77 | 0.54 ± 0.15 | 28.19 |
| Dentate nucleus | ns | 0.57 ± 0.07 | 11.89 | 0.55 ± 0.14 | 26.14 | 0.56 ± 0.15 | 27.40 |
| Subthalamic nucleus | ns | 0.56 ± 0.07 | 11.74 | 0.57 ± 0.14 | 23.85 | 0.57 ± 0.20 | 35.17 |
| Putamen | ns | 0.56 ± 0.09 | 16.22 | 0.57 ± 0.16 | 28.19 | 0.58 ± 0.18 | 30.59 |
| Substantia nigra | ns | 0.55 ± 0.08 | 14.28 | 0.50 ± 0.10 | 19.29 | 0.53 ± 0.14 | 27.26 |
| Dorsal midbrain | ns | 0.46 ± 0.06 | 12.02 | 0.44 ± 0.13 | 30.34 | 0.47 ± 0.13 | 26.63 |
| **Braak regions** |  | | | | | | |
| Braak I | + | 0.49 ± 0.06 | 12.52 | 0.46 ± 0.11 | 23.17 | 0.58 ± 0.16 | 27.26 |
| Braak II | ns | 0.50 ± 0.07 | 14.10 | 0.46 ± 0.10 | 21.06 | 0.51 ± 0.13 | 25.47 |
| Braak III | ns | 0.49 ± 0.06 | 13.09 | 0.46 ± 0.12 | 24.93 | 0.57 ± 0.20 | 34.51 |
| Braak IV | ns | 0.49 ± 0.06 | 13.05 | 0.46 ± 0.11 | 24.41 | 0.56 ± 0.17 | 31.02 |
| Braak V | ## + | 0.51 ± 0.06 | 12.25 | 0.51 ± 0.13 | 25.17 | 0.67 ± 0.20 | 29.12 |
| Braak VI | ns | 0.50 ± 0.06 | 12.76 | 0.49 ± 0.11 | 23.22 | 0.58 ± 0.16 | 27.31 |
|  |  | **V_T_ ratio (mean±SD)** | **CoV** | **V_T_ ratio (mean±SD)** | **CoV** | **V_T_ ratio (mean±SD)** | **CoV** |
| **PSP target regions** |  | | | | | | |
| MPFC | ns | 0.96 ± 0.08 | 8.13 | 0.96 ± 0.09 | 9.57 | 1.03 ± 0.17 | 16.27 |
| DLPFC | ## + | 1.01 ± 0.08 | 8.33 | 1.05 ± 0.06 | 5.54 | 1.20 ± 0.24 | 20.14 |
| Cerebellum | ns | 1.03 ± 0.02 | 2.06 | 1.01 ± 0.03 | 2.68 | 1.01 ± 0.02 | 2.06 |
| Globus pallidus | * + | 1.18 ± 0.10 | 8.78 | 1.32 ± 0.09 | 7.17 | 1.16 ± 0.09 | 7.74 |
| Globus pallidus externus | + | 1.19 ± 0.11 | 8.95 | 1.32 ± 0.10 | 7.20 | 1.17 ± 0.09 | 7.89 |
| Globus pallidus internus | * ++ | 1.16 ± 0.10 | 8.59 | 1.32 ± 0.10 | 7.68 | 1.14 ± 0.09 | 7.71 |
| Dentate nucleus | ns | 1.21 ± 0.06 | 5.21 | 1.22 ± 0.05 | 4.44 | 1.17 ± 0.04 | 3.24 |
| Subthalamic nucleus | * | 1.20 ± 0.07 | 5.61 | 1.29 ± 0.07 | 5.32 | 1.19 ± 0.11 | 9.20 |
| Putamen | ns | 1.21 ± 0.12 | 10.22 | 1.31 ± 0.08 | 5.87 | 1.22 ± 0.09 | 7.18 |
| Substantia nigra | ns | 1.17 ± 0.06 | 5.40 | 1.12 ± 0.12 | 10.76 | 1.11 ± 0.08 | 7.15 |
| Dorsal midbrain | ns | 0.98 ± 0.06 | 5.61 | 0.97 ± 0.10 | 9.99 | 0.99 ± 0.06 | 6.00 |
| **Braak regions** |  | | | | | | |
| Braak I | #### ++++ | 1.00 ± 0.06 | 5.69 | 1.00 ± 0.06 | 6.32 | 1.22 ± 0.06 | 5.21 |
| Braak II | # + | 1.03 ± 0.06 | 5.63 | 1.00 ± 0.09 | 9.35 | 1.09 ± 0.04 | 4.02 |
| Braak III | #### ++++ | 1.01 ± 0.07 | 7.05 | 1.00 ± 0.08 | 7.85 | 1.19 ± 0.11 | 9.54 |
| Braak IV | #### ++++ | 0.91 ± 0.05 | 5.90 | 0.89 ± 0.06 | 6.52 | 1.07 ± 0.10 | 9.31 |
| Braak V | ### +++ | 1.05 ± 0.06 | 6.02 | 1.09 ± 0.07 | 6.24 | 1.44 ± 0.32 | 21.85 |
| Braak VI | * # + | 1.01 ± 0.06 | 5.43 | 1.06 ± 0.07 | 6.49 | 1.24 ± 0.25 | 20.20 |

Supplemental Table 4: Comparison of regional mean V_T_ [ml/ccm] and V_T_ ratio [-] values with coefficients of variation (CoV) [%] for healthy controls (n = 15), PSP patients (n = 15) and AD patients (n = 10) generated by the manual method with five highest voxel intensity values (max5); significant differences are presented as follows: * p < 0.05, ** p < 0.01, *** p < 0.001, **** p < 0.0001; * healthy controls vs. PSP, # healthy controls vs. AD, + AD vs. PSP

|  | **SUV ratio** | **V_T_ ratio** | **DV ratio** |
| --- | --- | --- | --- |
| **PSP target regions** |  | | |
| MPFC | 0.39 (**) | 0.32 (*) | 0.24 (ns) |
| DLPFC | 0.49 (***) | 0.36 (**) | 0.40 (**) |
| Globus Pallidus | 0.51 (****) | 0.39 (**) | 0.35 (*) |
| Globus Pallidus externus | 0.53 (****) | 0.36 (**) | 0.35 (*) |
| Globus Pallidus internus | 0.48 (***) | 0.40 (**) | 0.32 (*) |
| Nucleus Dentatus | 0.42 (**) | 0.29 (*) | 0.17 (ns) |
| Nucleus Subthalamicus | 0.49 (***) | 0.42 (**) | 0.40 (**) |
| Putamen | 0.60 (****) | 0.38 (**) | 0.33 (*) |
| Substantia Nigra | 0.48 (***) | 0.39 (**) | 0.33 (*) |
| Dorsal midbrain | 0.52 (****) | 0.60 (****) | 0.27 (ns) |
| **Braak regions** |  | | |
| Braak I | 0.49 (***) | 0.42 (**) | 0.43 (**) |
| Braak II | 0.37 (**) | 0.25 (ns) | 0.31 (*) |
| Braak III | 0.56 (****) | 0.53 (****) | 0.47 (***) |
| Braak IV | 0.52 (****) | 0.46 (***) | 0.46 (***) |
| Braak V | 0.65 (****) | 0.60 (****) | 0.59 (****) |
| Braak VI | 0.49 (***) | 0.43 (**) | 0.44 (**) |

Supplemental Table 5: Pearson correlation coefficients [-] of healthy controls (n = 15), patients with PSP (n = 15) and patients with AD (n = 10) for V_T_ values [ml/ccm] and V_T_ ratio [-], SUV ratio [-] and DV ratio [-] values in PSP target regions and Braak regions (* p < 0.05, ** p < 0.01, *** p < 0.001, **** p < 0.0001)
